# Coordinated inheritance of extrachromosomal DNAs in cancer cells

**DOI:** 10.1038/s41586-024-07861-8

**Published:** 2024-11-06

**Authors:** King L. Hung, Matthew G. Jones, Ivy Tsz-Lo Wong, Ellis J. Curtis, Joshua T. Lange, Britney Jiayu He, Jens Luebeck, Rachel Schmargon, Elisa Scanu, Lotte Brückner, Xiaowei Yan, Rui Li, Aditi Gnanasekar, Rocío Chamorro González, Julia A. Belk, Zhonglin Liu, Bruno Melillo, Vineet Bafna, Jan R. Dörr, Benjamin Werner, Weini Huang, Benjamin F. Cravatt, Anton G. Henssen, Paul S. Mischel, Howard Y. Chang

**Affiliations:** 1 Center for Personal Dynamic Regulomes, Stanford University, Stanford, CA USA; 2 Sarafan ChEM-H, Stanford University, Stanford, CA USA; 3 Department of Pathology, Stanford University, Stanford, CA USA; 4 School of Medicine, University of California at San Diego, La Jolla, CA USA; 5 Department of Computer Science and Engineering, University of California at San Diego, La Jolla, CA USA; 6 Experimental and Clinical Research Center (ECRC), Max Delbrück Center for Molecular Medicine and Charité—Universitätsmedizin Berlin, Berlin, Germany; 7 Department of Pediatric Oncology/Hematology, Charité—Universitätsmedizin Berlin, Berlin, Germany; 8 Department of Mathematics, Queen Mary University of London, London, UK; 9 Max-Delbrück-Centrum für Molekulare Medizin (BIMSB/BIH), Berlin, Germany; 10 Department of Chemistry, Scripps Research, La Jolla, CA USA; 11 Chemical Biology and Therapeutics Science Program, Broad Institute, Cambridge, MA USA; 12 Evolutionary Dynamics Group, Centre for Cancer Genomics and Computational Biology, Barts Cancer Institute, Queen Mary University of London, London, UK; 13 Group of Theoretical Biology, The State Key Laboratory of Biocontrol, School of Life Science, Sun Yat-sen University, Guangzhou, China; 14 Vividion Therapeutics, San Diego, CA USA; 15 German Cancer Consortium (DKTK), partner site Berlin and German Cancer Research Center DKFZ, Heidelberg, Germany; 16 Berlin Institute of Health, Berlin, Germany; 17 Department of Genetics, Stanford University, Stanford, CA USA; 18 Howard Hughes Medical Institute, Stanford University School of Medicine, Stanford, CA USA

**Keywords:** Tumour heterogeneity, Cancer genetics, Genome, Cytogenetics, Cell division

## Abstract

The chromosomal theory of inheritance dictates that genes on the same chromosome segregate together while genes on different chromosomes assort independently^1^. Extrachromosomal DNAs (ecDNAs) are common in cancer and drive oncogene amplification, dysregulated gene expression and intratumoural heterogeneity through random segregation during cell division^2,3^. Distinct ecDNA sequences, termed ecDNA species, can co-exist to facilitate intermolecular cooperation in cancer cells^4^. How multiple ecDNA species within a tumour cell are assorted and maintained across somatic cell generations is unclear. Here we show that cooperative ecDNA species are coordinately inherited through mitotic co-segregation. Imaging and single-cell analyses show that multiple ecDNAs encoding distinct oncogenes co-occur and are correlated in copy number in human cancer cells. ecDNA species are coordinately segregated asymmetrically during mitosis, resulting in daughter cells with simultaneous copy-number gains in multiple ecDNA species before any selection. Intermolecular proximity and active transcription at the start of mitosis facilitate the coordinated segregation of ecDNA species, and transcription inhibition reduces co-segregation. Computational modelling reveals the quantitative principles of ecDNA co-segregation and co-selection, predicting their observed distributions in cancer cells. Coordinated inheritance of ecDNAs enables co-amplification of specialized ecDNAs containing only enhancer elements and guides therapeutic strategies to jointly deplete cooperating ecDNA oncogenes. Coordinated inheritance of ecDNAs confers stability to oncogene cooperation and novel gene regulatory circuits, allowing winning combinations of epigenetic states to be transmitted across cell generations.

## Abstract (web summary)

Cooperative species of extrachromosomal DNAs are coordinately inherited through mitotic co-segregation.

## Main

Oncogene amplification drives cancer development by increasing the copies of genetic sequences that encode oncogene products. Oncogenes are frequently amplified on megabase-sized circular ecDNA, which is detected in half of human cancer types^5^. First reported in 1965 (ref. ^6^), ecDNA amplifications (also known as double minutes^7^) have been shown to promote cancer development by driving copy-number heterogeneity^5,8^ and rapid adaptation to selective pressure in cancer^9–11^. This heterogeneity and adaptability can be attributed to the fact that, although ecDNA is replicated in each cell cycle and transmitted through cell division, owing to their lack of centromeres, ecDNA molecules are inherited unevenly among daughter cells during mitosis^12–14^.

ecDNAs exhibit a substantial level of genetic sequence diversity. First, multiple ecDNAs originally derived from different chromosomal loci can co-exist in the same cancer cell, often congregating in micrometre-sized hubs in the nucleus that enable intermolecular gene activation between distinct ecDNAs^4,15^. Second, ecDNAs contain clustered somatic mutations that suggest APOBEC3-mediated mutagenesis^16^, increasing the diversity of ecDNA sequence and function^16–18^. Third, ecDNAs can contain complex structural rearrangements of sequences originating from various genomic sites^4,11,18–21^. DNA damage can cause ecDNAs to cluster and sometimes become incorporated into micronuclei^7,22–24^, where DNA can further fragment and recombine^25–27^. These rearrangement events can give rise to diverse, co-existing ecDNA species in a cell population, including ecDNAs with distinct oncogene loci^4,11,18,20,28^ or encompassing only enhancers or oncogene coding sequences^18^.

Observations of diverse ecDNA species co-occurring in the same cell containing distinct oncogenes^4,11,18,20^ suggest that ecDNAs may represent specialized, cooperative molecules. For example, it has been reported that new ecDNA species can form in cells after recurrence or drug treatment of ecDNA-carrying cancers while the original ecDNA amplicons are retained^11,29^, suggesting that multiple ecDNA species may arise independently and that their interaction provides fitness advantages to cancer cells. Concordantly, ecDNAs carrying oncogenes alongside non-coding regulatory elements can interact with each other and with chromosomes in a combinatorial manner to promote gene expression^3,4,30^. These observations lend support to the hypothesis that the co-occurrence of multiple ecDNA sequences in a cell may have combinatorial and synergistic effects on transcriptional programs.

The diversity of ecDNA genetic sequences and importance of intermolecular interactions between ecDNAs in a cancer cell population raises the questions of (1) how heterogeneous ecDNA species are distributed in a cell population; (2) as ecDNAs are segregated unequally during mitosis, how these mixtures of ecDNAs are inherited by daughter cells; and (3) how the dynamics of multiple ecDNA species affect cancer evolution under selective pressure. Using a combination of image analysis, single-cell and bulk sequencing, and computational modelling, we set out to elucidate the principles and consequences of ecDNA co-evolution in cancer.

## Distinct ecDNAs co-occur in cancer cells

**Fig. 1:**
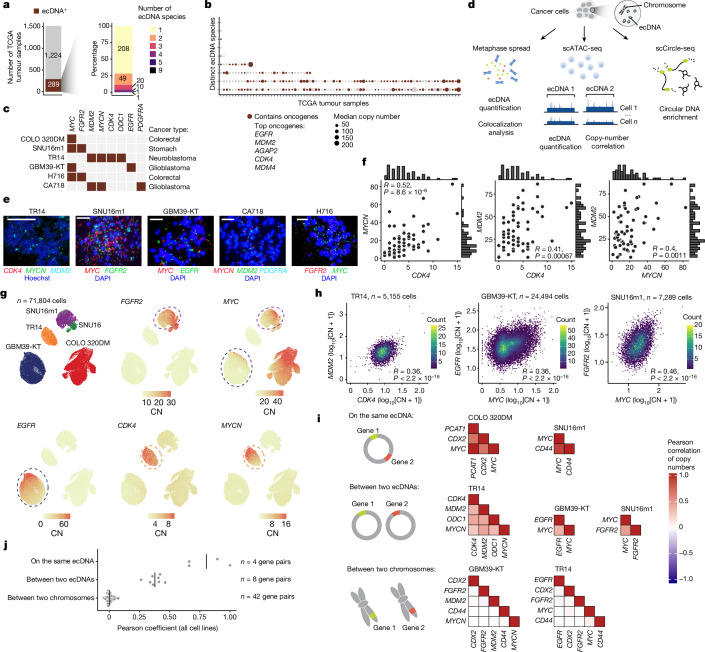
ecDNA species encoding distinct oncogene sequences are correlated in individual cancer cells. **a**, Summary of ecDNA-positive tumours (left) and the number of ecDNA species (right) identified in TCGA tumour samples. **b**, The median copy numbers and oncogene statuses of distinct ecDNA species in TCGA tumours that were identified to have more than one ecDNA species. **c**, A panel of cell lines with known oncogene sequences on ecDNA. **d**, Schematic of the ecDNA analyses using three orthogonal approaches: metaphase spread, scATAC-seq and scCircle-seq. **e**, Representative DNA-FISH images of metaphase spreads with FISH probes targeting various oncogene sequences as indicated. *n* = 64 (TR14), *n* = 76 (SNU16m1), *n* = 62 (GBM39-KT), *n* = 70 (CA718) and *n* = 82 (H716) cells. Scale bars, 10 µm. **f**, Oncogene copy-number scatter plots and histograms of pairs of oncogenes in TR14 cells. Statistical analysis was performed using two-sided Spearman correlation. **g**, Uniform manifold approximation and projection (UMAP) analysis of scATAC-seq data showing cell line annotations and copy-number (CN) calculations of indicated oncogenes. **h**, The log-transformed oncogene copy numbers between pairs of oncogenes in the indicated cell lines (Pearson’s *R*, two-sided test; *P* < 2.2 × 10^−16^ for all correlations). **i**, Pearson correlation heat maps of gene pairs on the same ecDNA (top), between two ecDNAs (middle) and between two chromosomes (bottom). **j**, Pearson correlation coefficients of gene pairs on the same ecDNA (*n* = 4 gene pairs), between two ecDNAs (*n* = 8 gene pairs) and between two chromosomes (*n* = 42 gene pairs) in COLO 320DM, SNU16, SNU16m1, TR14 and GBM39-KT cells.

**Extended Data Fig. 1:**
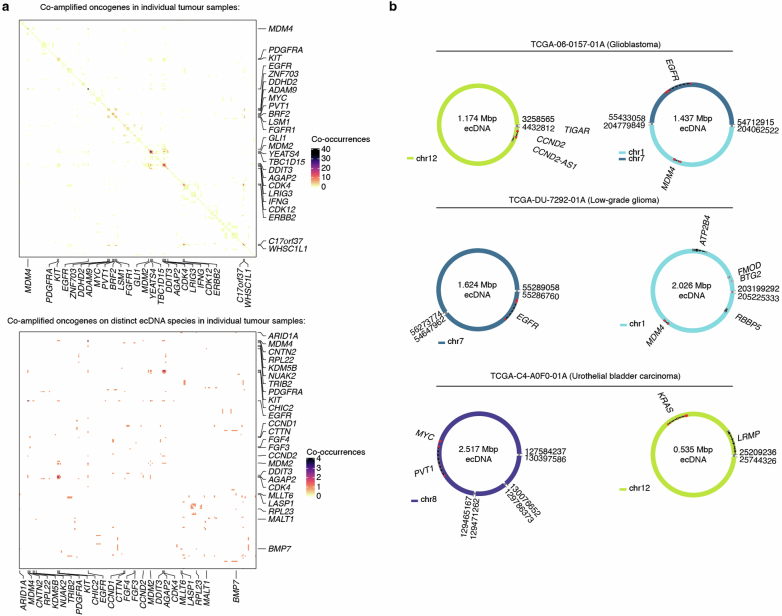
Oncogenes on co-occurring ecDNA species in TCGA samples. **(a)** Heatmaps showing co-occurrences of ecDNA-amplified oncogenes in individual tumour samples (top) and co-occurrences of oncogenes on distinct ecDNA species in individual tumour samples (bottom). Genes are sorted based on their chromosomal locations. **(b)** Examples of reconstructions of co-occurring, distinct ecDNA species in a glioblastoma sample, a low-grade glioma sample, and a urothelial bladder carcinoma sample.

To examine how frequently ecDNA molecules with distinct sequences co-exist in the same tumours, we first analysed ecDNA structures predicted from whole-genome sequencing (WGS) data in The Cancer Genome Atlas^19^ (TCGA; Methods). This analysis revealed that 289 out of 1,513 patient tumours contained ecDNA, carrying coding sequences of well-characterized oncogenes such as *EGFR*, *MDM2* and *CDK4* (refs. ^5,19^) (Fig. 1a,b). Among tumours that contained ecDNA, more than 25% (81 samples) contained two or more ecDNA species in the same tumour (Fig. 1a and Extended Data Fig. 1a). Many of these ecDNA species were highly amplified and contained canonical oncogenes (Fig. 1b), supporting the idea that heterogeneous ecDNA sequences can be found in the same tumour and their co-occurrence may provide distinct selective advantages (such as *CCND2*, *EGFR* and *MDM4* in a glioblastoma sample, and *MYC* and *KRAS* in a urothelial bladder carcinoma sample; Extended Data Fig. 1b). As we considered only highly abundant and genomically non-overlapping ecDNA sequences as distinct species, this analysis probably underestimates the true diversity of ecDNA species.

**Extended Data Fig. 2:**
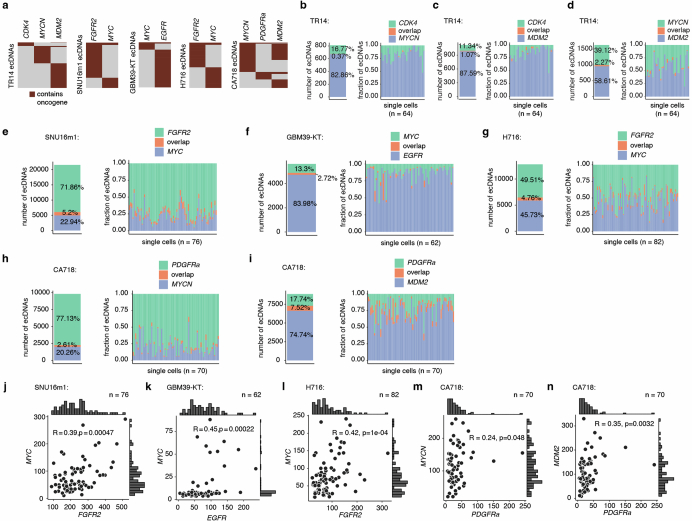
Oncogenes are harboured on distinct ecDNA species but are correlated in copy number. **(a)** Heatmaps showing non-overlapping oncogene presence on distinct ecDNA species in metaphase DNA FISH. Rows represent individual ecDNA molecules. **(b-d)** Bar plots showing the fractions of ecDNAs containing combinations of *MYCN*, *CDK4* or *MDM2* and demonstrating little overlap between these oncogenes on the same ecDNA molecules. **(e-n)** Covalent linkage and copy-number correlations between distinct ecDNA species in metaphase DNA FISH images of various cell lines. Bar plots showing the fractions of ecDNAs containing combinations of *FGFR2* and *MYC* in SNU16m1 cells **(e)**, *MYC* and *EGFR* in GBM39-KT cells **(f)**, *MYC* and *FGFR2* in H716 cells **(g)**, and *MYCN*, *PDGFRa* and *MDM2* in CA718 cells **(h,i)**. Copy number correlations and distributions of *FGFR2* and *MYC* ecDNAs in SNU16m1 cells **(j)**, *MYC* and *EGFR* in GBM39-KT cells **(k)**, *MYC* and *FGFR2* in H716 cells **(l)**, and *MYCN*, *PDGFRa* and *MDM2* in CA718 cells **(m,n)**. Spearman correlations, two-sided test in (**j-n**).

The frequent co-amplification of distinct ecDNA species in tumours raised the question of whether multiple ecDNA species can co-occur in the same cells. We examined a panel of cancer cell line and neurosphere models that were previously characterized to contain multiple ecDNA species^4,5,9^ (Fig. 1c). After validating each cell line using DNA fluorescence in situ hybridization (FISH) analysis of metaphase chromosome spreads (Fig. 1d,e), we found that the vast majority of individual cells had very little overlap in FISH signals from distinct oncogenes on chromosome spreads (ranging from 2–7%; Fig. 1e and Extended Data Fig. 2a–i). These data confirmed that distinct ecDNAs are not covalently linked on the same ecDNA molecule and are therefore expected to be inherited independently from one another in dividing cancer cells.

**Extended Data Fig. 3:**
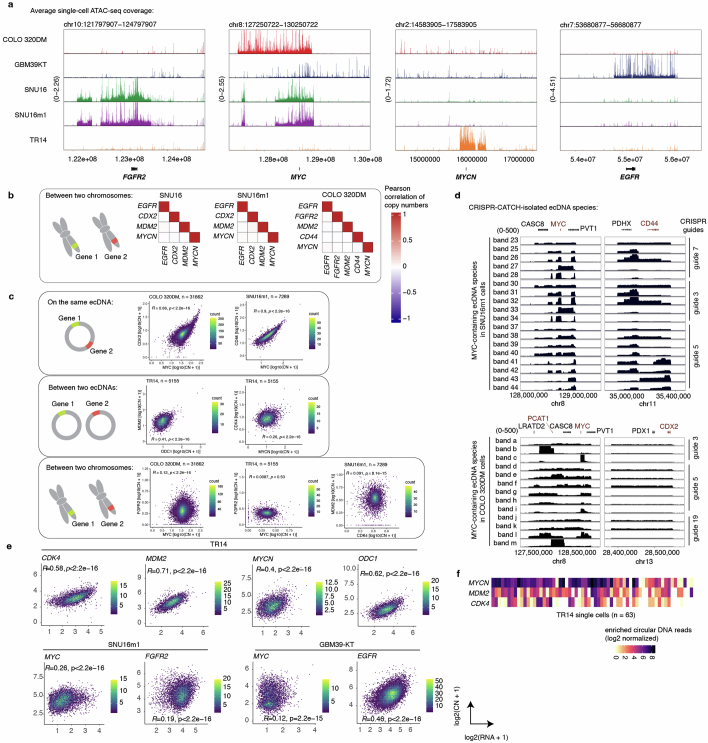
Distinct ecDNA amplifications co-occur and correlate at the single-cell level and their copy numbers affect transcriptional outcomes of oncogenes. **(a)** Elevated scATAC-seq background coverages of oncogene loci in correspondence to ecDNA copy number amplification in the various indicated cell lines. **(b)** Pearson correlation heatmaps of gene pairs between two chromosomes. **(c)** Density scatter plots showing levels of copy number correlation between gene pairs on the same ecDNA, on different ecDNAs, and on different chromosomes. **(d)** Sequencing coverages of ecDNA species isolated by CRISPR-CATCH from SNU16m1 cells and COLO 320DM cells, identifying genes that are frequently linked on the same ecDNA species (Methods). Each row represents a distinct ecDNA species isolated by molecular size fractionation using CRISPR-CATCH. Gene annotations in red are gene pairs classified as being on the same ecDNA in Fig. 1. All guide sequences are provided in Supplementary Table 1. **(e)** Density scatter plots showing correlation between oncogene copy number and RNA expression in paired scATAC-seq and RNA-seq. Cells with zero values were filtered. **(f)** Heatmap showing co-enrichment of circular DNA species containing *MYCN*, *MDM2* or *CDK4* in individual TR14 neuroblastoma cells in scCircle-seq.

We next examined the distributions of ecDNA copy numbers in single cells using three orthogonal methods (Fig. 1d): (1) metaphase chromosome spreading followed by DNA-FISH; (2) isolation of single nuclei followed by droplet-based single-cell assay for transposase-accessible chromatin using sequencing (scATAC-seq) and RNA sequencing (RNA-seq); and (3) enrichment and sequencing of ecDNAs in individual cells through exonuclease digestion and rolling circle amplification^31^ (scCircle-seq; Methods). Notably, in cell lines with distinct ecDNA species, FISH imaging revealed that pairs of ecDNA species had significantly correlated copy numbers (Spearman correlation *R* = 0.24–0.52, *P* < 0.05 in all cases; Fig. 1f and Extended Data Fig. 2j–n). We next assessed the significance of these correlations in a larger population of 71,804 cells from a subpanel of cell lines by adapting a copy-number quantification method for genomic background coverage from scATAC-seq data^4,32,33^ to calculate ecDNA copy numbers (Fig. 1d,g, Methods and Extended Data Fig. 3a). Notably, we observed positive correlations between distinct ecDNA species in each of the three cell lines with multiple ecDNA species (Fig. 1h–j and Extended Data Fig. 3b,c; Pearson correlation, *R* = 0.26–0.46, *P* < 1 × 10^−15^ in all cases). As expected, genic sequences that are covalently linked on the same ecDNA molecule (as demonstrated by isolation from the same molecular size fractions using CRISPR–CATCH^18^; Extended Data Fig. 3d) showed strong copy-number correlation in this analysis, validating this approach for measuring the distributions of ecDNA molecules in a cell population (Fig. 1i,j and Extended Data Fig. 3b,c). ecDNA copy numbers were positively correlated with RNA expression of the correspondingly amplified oncogenes, supporting the idea that the copies of ecDNA species drive transcriptional outcomes (Extended Data Fig. 3e). Importantly, we did not observe copy-number correlations between gene pairs located on different chromosomes, suggesting that this relationship between different ecDNA species cannot simply be explained by sequencing quality (Fig. 1i,j and Extended Data Fig. 3b,c). Finally, single-cell Circle-seq confirmed co-enrichment of the *MYCN*, *MDM2* and *CDK4* ecDNA species in individual TR14 neuroblastoma cells (Extended Data Fig. 3f).

**Extended Data Fig. 4:**
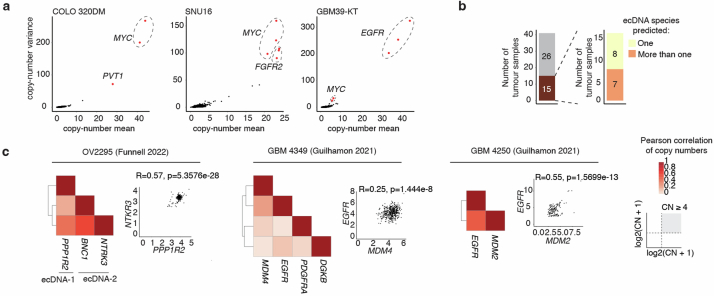
Inferring ecDNA amplifications and co-occurrence from single-cell copy-number data. **(a)** Mean and variance of copy-number distribution of 3-Mb genomic windows in three cell lines with validated ecDNA amplifications. Intervals with mean copy-number ≥ 4 and variance/mean ratio ≥ 2.5 were predicted as carrying ecDNA and highlighted in red. Known ecDNA amplifications are annotated onto predicted ecDNA intervals. **(b)** Number of samples predicted as carrying one or more ecDNA species in three public scATAC-seq and scDNA-seq datasets. **(c)** Pearson correlation heatmaps and representative scatter plots for samples predicted to carry more than one ecDNA species in (b). Two-sided p-values are reported for Pearson correlations. Correlations are reported across genes predicted to be on ecDNA, only considering CN ≥4 to focus on cells with co-amplification, and log2(1+x) copy numbers are reported for representative scatter plots.

To investigate whether patient tumours with variable oncogene copy numbers exhibit a similar signature of copy-number correlation in single cells, we curated a dataset of 41 tumour samples from publicly available scATAC-seq or single-cell DNA-seq data of triple-negative breast cancer, high-grade serous ovarian cancer and glioblastoma^34–36^. We devised a statistical approach for identifying focal amplifications using single-cell copy-number profiles and validated our ability to identify ecDNA amplifications in well-characterized cell lines (Methods and Extended Data Fig. 4a). Applying this approach to patient tumours, we found that 15 out of 41 (37%) cases had focal amplifications matching the signature of ecDNA. We further predicted 7 cases (17% of all samples) with focal amplification of two or more oncogenes with significantly correlated copy numbers in single cells, suggestive of co-amplified distinct ecDNA species (Extended Data Fig. 4b,c).

Together, these results show that distinct ecDNA species tend to co-occur with correlated copy numbers far more than expected by chance both in cancer cell lines and patient samples.

## Distinct ecDNA species co-segregate

**Fig. 2:**
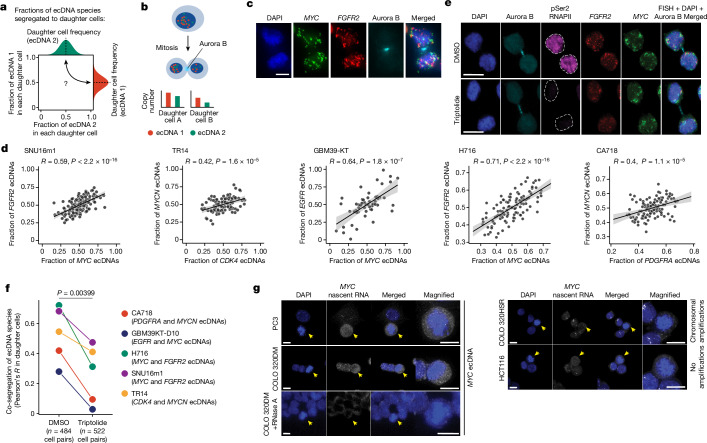
Distinct ecDNA species are co-segregated into daughter cells during mitosis. **a**, Individual ecDNA species are randomly inherited by daughter cells but their joint inheritance is unknown. **b**, Daughter cell pairs undergoing mitosis were identified by immunofluorescence for Aurora kinase B (Aurora B). Individual ecDNAs were quantified using sequence-specific FISH probes. **c**, Representative images of pairs of SNU16m1 daughter cells undergoing mitosis. *n* = 164 cells. Scale bars, 5 µm. **d**, Per-cell ecDNA contents in daughter cells of cancer cell lines (two-sided Pearson’s *R*; SNU16m1, *P* < 2.2 × 10^−16^; TR14, *P* = 1.6 × 10^−5^; GBM39-KT, *P* = 1.8 × 10^−7^; H716, *P* < 2.2 × 10^−16^; CA718, *P* = 1.1 × 10^−5^). H716 and CA718 were treated with DMSO for 3.5 h. The error bands represent the 95% confidence intervals. **e**, Representative images of immunofluorescence–DNA-FISH staining for Aurora kinase B protein, marking dividing daughter cells and active RNA polymerase II with serine 2 phosphorylation (pSer2 RNAPII), and *FGFR2* and *MYC* ecDNA in SNU16m1 cells treated with 10 µM triptolide (*n* = 206 cell pairs) or DMSO control (*n* = 177 cell pairs) for 3.5 h. The white dashed line indicates the nuclear boundary. Scale bars, 10 µm. **f**, Co-segregation of ecDNA species (Pearson’s *R*) in DMSO (control) and triptolide (10 µM) treatments for 3.5 h across cancer cell lines. *P* values were calculated using one-sided Fisher’s *z*-transformation for both individual cell lines and paired *t*-test for all cell lines. **g**, Representative images of intron RNA-FISH images detecting *MYC* intron 2 as a readout for nascent transcription in cell lines with *MYC* amplified on ecDNA (PC3, COLO 320DM), chromosomes (COLO 320HSR) or no *MYC* amplification (HCT116). *n* = 37 (PC3), *n* = 37 (COLO 320DM), *n* = 19 (COLO 320DM with RNase A), *n* = 41 (COLO 320HSR) and *n* = 38 (HCT116) cells. An RNase-A-treated negative control shows loss of intron RNA-FISH signal. The yellow arrows indicate mitotic cells with condensed chromatin. Scale bars, 10 µm.

**Extended Data Fig. 5:**
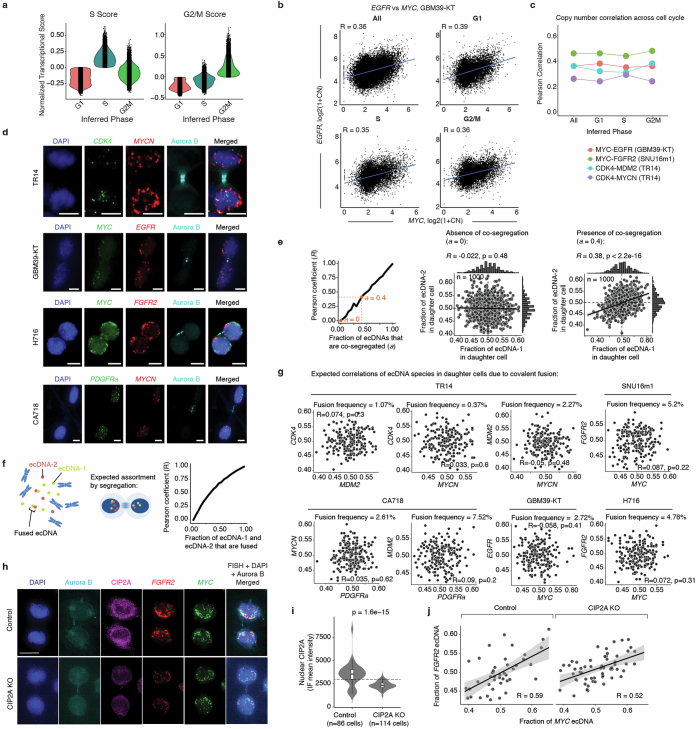
ecDNA co-inheritance is not explained by hyper-replication, covalent fusion, or CIP2A mitotic tethering. **(a)** Distribution of S phase and G2M phase transcriptional signatures across inferred cell cycle phases across cells profiled with paired scATAC-seq and RNA-seq (n = 71,804 cells). **(b)** Scatter plot of *MYC* and *EGFR* copy-number correlations overall and across inferred cell phases for GBM39-KT. Each dot is a single cell; Pearson correlations are reported for each grouping. **(c)** Summary of copy-number Pearson correlations for pairs of ecDNA genes in GBM39-KT, SNU16m1, and TR14 overall and across cell cycle phases. **(d)** Representative images of pairs of daughter cells undergoing mitosis. Scale bars, 5 µm. **(e)** Segregation of two ecDNA species with 100 copies each was simulated by random sampling with varying levels of co-segregation (1000 simulations per co-segregation fraction *a*; Methods). As the fraction (*a*) of ecDNAs that are co-segregated increases from 0.00 (no co-segregation) to 1.00 (each copy of one ecDNA species is perfectly co-segregated with a copy of another species) in increments of 0.05, the Pearson coefficient *R* of the copy numbers of two ecDNA species in individual daughter cells increases linearly (left panel). Thus, in the absence of co-segregation, no copy number correlation in mitotic daughter cells is expected (middle panel), while in the presence of a modest level of co-segregation (a fraction of 0.4, or 40% of one ecDNA species co-segregating with 40% of another), a Pearson coefficient *R* of 0.38 is expected (right panel). Two-sided test was used to calculate significance. **(f)** Segregation of two ecDNA species with 100 copies each was simulated by random sampling with varying frequencies of covalent fusion (from 0.00 to 1.00 with increments of 0.05; 5000 simulations per fusion frequency; Methods). Left panel shows resulting Pearson’s R in dividing daughter cells explained by various levels of covalent fusion. **(g)** Expected copy number correlations between pairs of ecDNA species in dividing daughter cells in the indicated cancer cell lines based on quantified levels of covalent fusion (Pearson’s R, two-sided test). **(h)** Representative images of immunofluorescence-DNA-FISH staining for Aurora kinase B protein marking dividing daughter cells, CIP2A, *FGFR* and *MYC* ecDNA, and DNA staining by DAPI in SNU16m1 cells after treatment with CRISPR-Cas9 and a non-targeting control guide RNA or a guide RNA targeting the protein coding sequence of CIP2A (CIP2A KO). Scale bars, 10 µm. **(i)** Violin plot showing CIP2A levels in the control (n = 86 cells) or CIP2A KO (n = 114 cells) SNU16m1 cells. Box center line, median; box limits, upper and lower quartiles; box whiskers, 1.5× interquartile range. **(j)** Per-cell ecDNA contents containing the indicated oncogene sequences of daughter cells in the control or CIP2A KO SNU16m1 cells (Pearson’s R; error bands represent 95% confidence intervals). The difference between the two correlations is not statistically significant (Fisher’s z-transformation and one-sided test).

In principle, our observations of co-occurrence and correlation of two distinct ecDNA species can be the result of (1) hyper-replication of ecDNAs in a subpopulation of cells; (2) co-selection of both species, given that both species provide fitness advantages and/or engage in synergistic intermolecular interactions; or (3) co-segregation of both species into daughter cells during cell division. To investigate whether hyper-replication contributes to the observed ecDNA correlation, we evaluated copy-number correlations in cells across different phases of the cell cycle using the single-cell multi-omics data (Methods). We observed no additional co-enrichment of ecDNA in cells that have replicated their DNA (Extended Data Fig. 5a–c), which is consistent with previous literature reporting that ecDNA is replicated once per cell cycle, along with genomic DNA, during S phase^37,38^. Conversely, as different ecDNA species can carry different oncogenes and mixed ecDNAs can interact with each other to increase gene expression^4,30^, co-selection can reasonably explain co-occurrence of ecDNA species. However, given their stochastic segregation into daughter cells^12–14^, it is unclear how a collective of ecDNA species and their cooperative interactions are preserved over successive cell divisions (Fig. 2a).

To address this question, we assessed the distribution of multiple ecDNA species during a single cell division. Using DNA-FISH combined with immunofluorescence staining for Aurora kinase B, a component of the mitotic midbody, we quantified the copy numbers of ecDNA inherited among daughter cell pairs undergoing mitosis^12,39^ (Fig. 2b). Notably, in all five cancer cell lines containing multiple distinct ecDNA species (Fig. 1c,e and Extended Data Fig. 2), we observed significant co-segregation of distinct ecDNA species to daughter cells as measured by the correlated proportions of ecDNAs inherited (*R* = 0.4–0.71, *P* < 1 × 10^−4^ in each case; Fig. 2c,d, Methods and Extended Data Fig. 5d). In other words, the daughter cell that inherits more copies of ecDNA species 1 tends to inherit more copies of species 2, and vice versa. Simulations of segregating ecDNAs showed that this correlation of ecDNA species in daughter cells is far greater than expected from random segregation, or the levels of co-inheritance contributed by rare covalent fusions of ecDNAs, and scales linearly with the level of co-segregation of ecDNAs (Methods and Extended Data Fig. 5e–g). It is unlikely that this result would be driven by cellular volumetric differences as ecDNA segregate by colocalizing with mitotic chromosomes rather than spreading by diffusion^4,13,40^. Together, these data show that, while individual ecDNAs segregate into daughter cells following a binomial distribution^12,14^, collectives of ecDNA species may co-segregate during mitosis.

## Transcription promotes co-segregation

We next investigated the molecular mechanism of ecDNA co-segregation. Previous studies have shown that ecDNAs aggregate in response to artificially induced DNA damage^22,23^; more recent reports showed that damaged DNA fragments are tethered together in mitosis by the CIP2A–TOPBP1 complex and co-segregate^25,41^. However, CIP2A localizes to DNA breaks and does not to bind to intact ecDNAs^41^. Consistent with this report, we found that genetic knockout of *CIP2A* had no significant effect on co-segregation of ecDNA species (Extended Data Fig. 5h–j).

**Extended Data Fig. 6:**
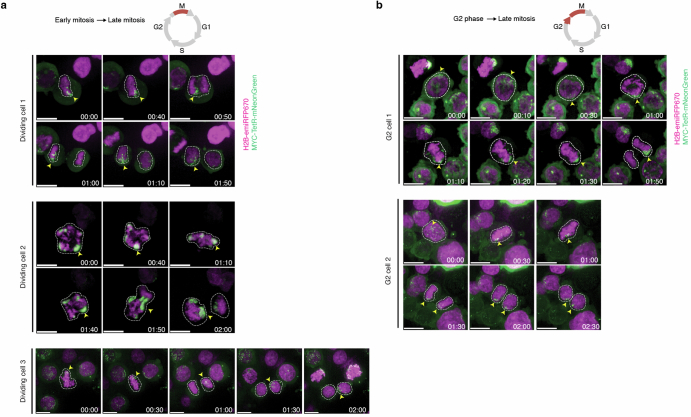
Live cell imaging of ecDNA localization during cell division. **(a-b)** Representative time-lapsed images of TetR-mNeonGreen labelled *TetO-MYC* ecDNAs in COLO 320DM cells undergoing early to late mitosis (3 dividing cell pairs) **(a)** and G2 to late mitosis (2 dividing cell pairs) **(b)** (n = 30 cell divisions over 5 independent time-lapse experiments). H2B-emiRFP670 labels histone H2B to aid identification of mitotic chromosomes and/or nuclear boundaries. White dash lines denote the mitotic cell across time frames; clumps of ecDNA molecules observed throughout mitosis are indicated with yellow arrowheads. Time stamp is denoted in hh:mm format. Scale bar = 10 μm.

**Extended Data Fig. 7:**
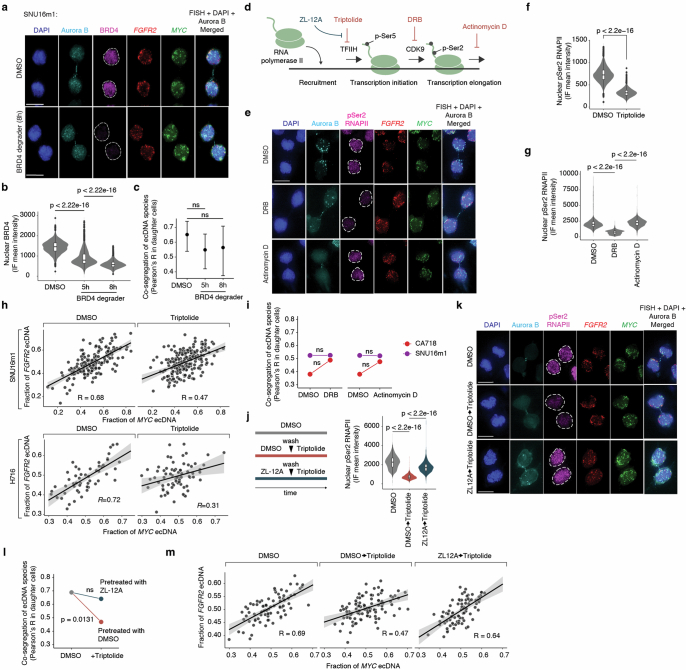
Active transcription initiation promotes coordinated inheritance of ecDNA species. **(a)** Representative image of immunofluorescence (IF)-DNA-FISH staining for simultaneous labelling of Aurora kinase B protein marking dividing daughter cells, BRD4 protein, *FGFR2* ecDNA and *MYC* ecDNA in SNU16m1 cells treated with a BRD4 degrader at 1 µM or DMSO (control) for 8 h. White dashed line marks the nuclear boundary. Scale bars, 10 µm. **(b-c)** BRD4 protein level and corresponding changes in co-segregation of ecDNA species upon BRD4 degrader treatment in SNU16m1 cells. **(b)** Violin plot showing nuclear BRD4 IF mean intensity scores of SNU16m1 interphase cells treated with DMSO (n = 2774 cells) or BRD4 degrader for 5 h (n = 2030 cells) or 8 h (n = 2338 cells). P-values computed with a two-sided Wilcoxon rank-sums test. Box center line, median; box limits, upper and lower quartiles; box whiskers, 1.5× interquartile range. **(c)** Levels of co-segregation of *FGFR2* and *MYC* ecDNA quantified by Pearson’s R between the two ecDNA species in dividing SNU16m1 daughter cells and the respective mean nuclear BRD4 IF intensities (DMSO, n = 128 cells; BRD4 degrader: 5 h, n = 139 cells; 8 h, n = 67 cells). Statistical significance was computed using Fisher’s z-transformation and one-sided hypothesis testing. Error bars show Zou’s 95% confidence intervals. ns, not significant. While the mean correlation coefficients are not statistically significant, the increased confidence interval with dBRD4 treatment suggests increased variance in ecDNA co-segregation. **(d)** A schematic diagram of transcription initiation and elongation which can be blocked by various chemical compounds. **(e)** Representative images of immunofluorescence-DNA-FISH staining for Aurora kinase B protein marking dividing daughter cells, active pSer2 RNAPII, *FGFR* and *MYC* ecDNA, and DNA staining by DAPI in SNU16m1 cells treated with DMSO (control), DRB (200 µg/mL) or actinomycin D (5 µg/mL) for 3 h. **(f)** Violin plot showing levels of active nuclear RNA Polymerase II with serine 2 phosphorylation (pSer2 RNAPII) in SNU16m1 interphase cells treated with DMSO (n = 3325 cells) or 10 µM triptolide (n = 2596 cells) for 3.5 h (p < 2.2e-16). P-value computed with a two-sided Wilcoxon rank-sums test. Box center line, median; box limits, upper and lower quartiles; box whiskers, 1.5× interquartile range. **(g)** Violin plot showing levels of active pSer2 RNAPII in SNU16m1 interphase cells treated with DMSO (n = 3401 cells), 200 µg/mL DRB (n = 1696 cells) or 5 µg/mL actinomycin D (n = 1371 cells) for 3 h (p < 2.2e-16 for DMSO vs DRB and DRB vs actinomycin D). P-values computed with a two-sided Wilcoxon rank-sums test. Box center line, median; box limits, upper and lower quartiles; box whiskers, 1.5× interquartile range. **(h)** Scatter plots showing per-cell ecDNA contents containing the indicated oncogene sequences of daughter cells in SNU16m1 and H716 after treatment with DMSO or 10 µM triptolide for 3.5 h (Pearson’s R; error bands represent 95% confidence intervals. DMSO-treated H716 data was also shown in Fig. 2d). **(i)** Pairwise comparisons of ecDNA co-segregation quantified by Pearson’s R between DMSO control and 200 µg/mL DRB or 5 µg/mL actinomycin D treatments for 3 h in SNU16m1 cells with *MYC* and *FGFR2* ecDNAs (DMSO, n = 86 daughter cell pairs; actinomycin D, n = 49 daughter cell pairs; DRB, n = 72 daughter cell pairs) or CA718 cells with *PDGFRa* and *MYCN* ecDNAs (DMSO, n = 60 daughter cell pairs; actinomycin D, n = 50 daughter cell pairs; DRB, n = 61 daughter cell pairs). Fisher’s z-transformation, one-sided test. **(j)** Left: Experimental schematic of cell treatments with DMSO or triptolide after pre-treatments with DMSO or ZL-12A, an antagonist of triptolide. Right: Violin plot showing levels of active pSer2 RNAPII in SNU16m1 interphase cells treated with DMSO (n = 1567 cells) for 6.5 h, or 10 µM triptolide for 3.5 h after pre-treatment of DMSO (n = 1467 cells) or 50 µM ZL-12A (n = 1135 cells) for 3 h. P-values computed with a two-sided Wilcoxon rank-sums test. Box center line, median; box limits, upper and lower quartiles; box whiskers, 1.5× interquartile range. **(k)** Representative images of immunofluorescence-DNA-FISH staining for Aurora kinase B protein marking dividing daughter cells, active pSer2 RNAPII, *FGFR* and *MYC* ecDNA, and DNA staining by DAPI in SNU16m1 cells with DMSO (n = 82 daughter cell pairs) for 6.5 h, or 10 µM triptolide for 3.5 h after pre-treatment of DMSO (n = 101 daughter cell pairs) or 50 µM ZL-12A (n = 81 daughter cell pairs) for 3 h. White dashed line marks the nuclear boundary. Scale bars, 10 µm. **(l)** Pairwise comparisons of ecDNA co-segregation quantified by Pearson’s R between DMSO for 6.5 h (n = 82 daughter cell pairs) and 10 µM triptolide for 3.5 h after pre-treatment with DMSO (n = 101 daughter cell pairs) or pre-treatment with 50 µM ZL-12A (n = 81 daughter cell pairs) for 3 h in SNU16m1 cells with *MYC* and *FGFR2* ecDNAs. Fisher’s z-transformation, one-sided test. **(m)** Per-cell ecDNA contents containing the indicated oncogene sequences of daughter cells in SNU16m1 after treatment with DMSO for 6.5 h or 10 µM triptolide for 3.5 h after pre-treatment with DMSO or pre-treatment with 50 µM ZL-12A for 3 h (Pearson’s R; error bands represent 95% confidence intervals).

As we and others have previously reported that different ecDNA species interact with one another through intermolecular contacts at transcriptionally active sites in ecDNA hubs during interphase^4,15^, we examined whether their co-segregation may be related to intermolecular proximity in the nucleus. To visualize ecDNA hubs during mitosis using live-cell imaging, we used the colorectal cancer COLO 320DM cell line with a Tet-operator (TetO) array inserted into *MYC* ecDNAs and fluorescently labelled ecDNA molecules using TetR-mNeonGreen (Methods). We observed in many cases that hubs of ecDNA molecules remained as a unit throughout mitosis, with many ecDNA molecules co-segregating into the same daughter nucleus (Extended Data Fig. 6a). Clusters of ecDNAs in G2 phase remained spatially proximal as cells entered mitosis, attached to the condensing chromosomes, and therefore co-segregated into the same daughter nucleus as a unit (Extended Data Fig. 6b). Inhibition of the bromodomain and extraterminal domain (BET) family of proteins has previously been shown to reduce ecDNA clustering^4^; while the level of ecDNA co-segregation showed a downward trend with BRD4 degradation (Methods), the effect was not significant, potentially due to incomplete degradation and compensatory effects by other members of the BET protein family (Extended Data Fig. 7a–c). To investigate the idea that intermolecular contacts at transcriptionally active sites may promote coordinated inheritance of ecDNA species, we next examined whether transcription inhibition can disrupt ecDNA co-segregation. We tested three different transcription inhibitors—triptolide, 5,6-dichlorobenzimidazole 1-β-d-ribofuranoside (DRB) and actinomycin D—targeting various steps of transcription initiation and elongation by RNA polymerase II^42–45^ (Fig. 2e and Extended Data Fig. 7d–g). We found that triptolide uniquely reduced ecDNA co-segregation in five cancer cell line models as measured by DNA-FISH and Aurora kinase B immunofluorescence imaging of late mitotic cells (*P* = 0.00399 for paired comparisons of all cell lines with triptolide treatments; in individual cell line comparisons, *P* < 0.05 in SNU16m1, CA718 and H716, and not significant in GBM39KT-D10 and TR14; DRB and actinomycin D had no effect on co-segregation in SNU16m1; Fig. 2f and Extended Data Fig. 7h,i). To further exclude potential off-target effects from triptolide, we pretreated cells with an antagonist of triptolide, ZL-12A, which induces the degradation of the transcription factor IIH (TFIIH) helicase ERCC3 by reacting with the same cysteine (Cys342) as triptolide, thereby attenuating triptolide-triggered degradation of RNA polymerase II^46^ (Extended Data Fig. 7d). Pretreatment with ZL-12A blocked the effects of triptolide on active RNA polymerase II as well as co-segregation of ecDNA species (Extended Data Fig. 7j–m), confirming the specific effect of transcription initiation on ecDNA co-segregation. As triptolide acts on transcription initiation through the TFIIH complex rather than elongation of RNA transcripts^45^ (Extended Data Fig. 7d), these results suggested that transcription initiation, but not transcription elongation, promotes ecDNA co-segregation. We observed this reduction of ecDNA co-segregation after only 3.5 h of triptolide treatment, suggesting that transcription inhibition very shortly before or during mitosis can disrupt ecDNA co-segregation. Consistent with this result, ecDNA remains transcriptionally active at the onset of mitosis, as shown by nascent oncogene RNA-FISH signal in ecDNA-containing cells at prometaphase but not when the same oncogene is located on chromosomes (Fig. 2g). Together, our live-cell imaging and chemical perturbation experiments support the idea that intermolecular proximity and active transcription before and at the start of mitosis facilitate the coordinated inheritance of ecDNA species into daughter cells.

## Modelling of ecDNA co-assortment

**Fig. 3:**
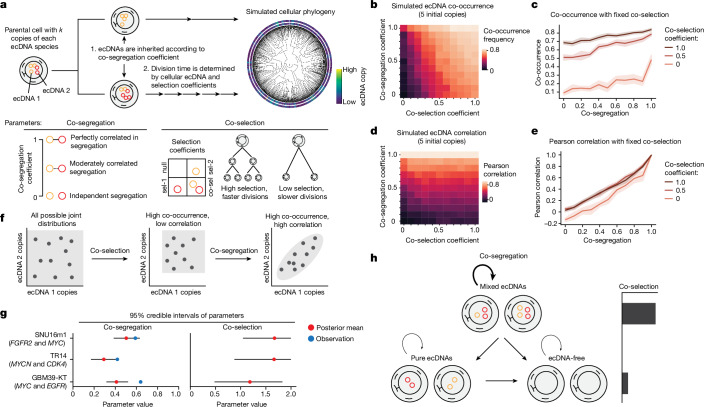
Evolutionary modelling of ecDNA dynamics reveals the principles of ecDNA co-inheritance. **a**, The evolutionary modelling framework used in this study. Cancer populations are simulated starting from a single parent cell carrying a user-defined set of distinct ecDNA species (here, we simulated 2 species) and user-defined initial copy numbers. Cells divide according to a fitness function, parameterized by user-defined selection coefficients. During cell division, ecDNA is inherited according to a co-segregation coefficient. **b**–**e**, Summary statistics of 1-million-cell populations and ten replicates across varying co-selection and co-segregation coefficients beginning with a parental cell with five copies of each ecDNA species. The average frequency of cells carrying both ecDNA species (**b**) and the Pearson correlation of ecDNA copy number within cells (**d**) are shown across all simulations. The mean frequency of cells carrying both ecDNA species (**c**) and Pearson correlation of ecDNA copy number within cells (**e**) are shown as a function of the co-segregation level for the following fixed levels of co-selection: 0.0, 0.5 and 1.0. The shaded area represents the 95% confidence interval across the experimental replicates. Selection acting on cells carrying one but not both ecDNAs is maintained at 0.2 and selection acting on cells without either ecDNA is maintained at 0.0 across all of the simulations. **f**, Schematic of the effects of co-selection and co-segregation on the joint distribution of ecDNA copy numbers in cancer cells. **g**, The 95% credible interval for inferred co-segregation and co-selection values for SNU16m1, TR14 and GBM39-KT cell lines. **h**, Conceptual summary of ecDNA co-evolutionary dynamics.

**Extended Data Fig. 8:**
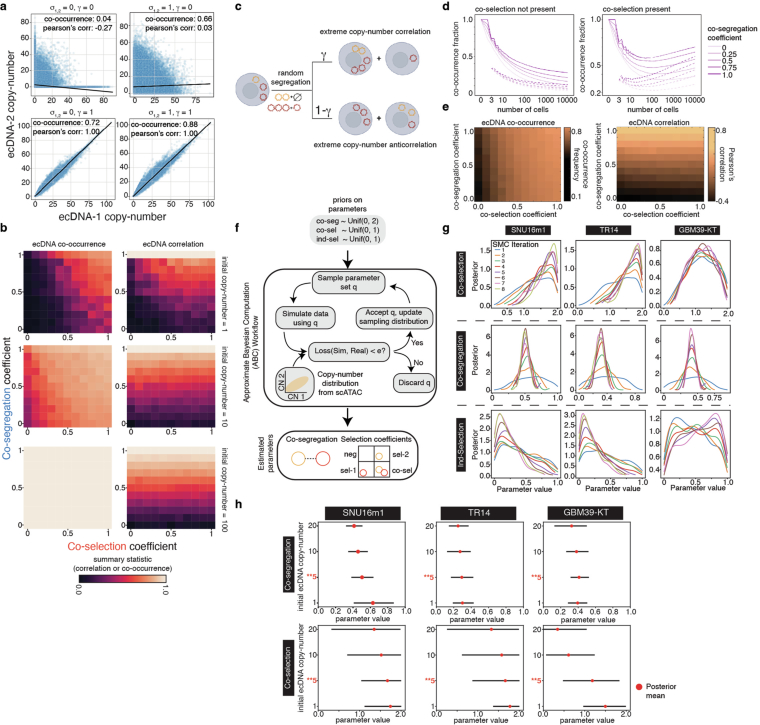
Additional analysis of evolutionary modelling of ecDNA. **(a)** Representative joint ecDNA copy-number distributions across varying levels of co-segregation and co-selection. Co-occurrence frequency and Pearson’s correlation are reported for each joint distribution. **(b)** Average frequency of cells carrying both ecDNA species and Pearson’s correlation of ecDNA copy-numbers in single cells are reported across simulations of 10 replicates of 1 million cells for varying initial ecDNA copy-numbers: 1 copy of each ecDNA species; 10 copies of each ecDNA species; 100 copies of each ecDNA species. Selection acting on cells with only one but not both ecDNA species is maintained at 0.2 and selection acting on cells without either ecDNA is maintained at 0.0 for all simulations. **(c)** A schematic illustrating an alternative model of ecDNA evolution, parameterized by selection acting on cells carrying no, both, or either ecDNA as well as a co-segregation parameter $\gamma$. **(d)** Frequency of cells carrying both ecDNA species reported as a function of number of cells during a simulation for variable levels of co-segregation and with or without co-selection. **(e)** Average frequencies of cells carrying both ecDNA species and the Pearson’s correlation of ecDNA copy numbers across 500 replicates of simulations of 10,000 cells while varying co-selection and co-segregation values. **(f)** Schematic of ABC inference workflow: posterior distributions over parameters are inferred from user-defined priors and observed single-cell copy-number data using sequential model fitting on our evolutionary model. **(g)** Posterior distributions of co-selection, co-segregation, and individual selection values for inferences in SNU16m1, TR14, and GBM39-KT across sequential iterations of Approximate Bayesian Inference Sequential Monte Carlo (ABC-SMC). **(h)** 95% credible interval of inferred co-segregation and co-selection values from ABC-SMC across the cell lines studied in this report with variable initial ecDNA copy numbers (1, 5, 10, 20). Mean of ABC-SMC-inferred posterior is reported for each 95% credible interval. The initial ecDNA copy number (5) used in the main text is highlighted in red. For simulations in (g-h) populations of 500,000 cells were simulated until the ABC-SMC procedure converged (target error of 0.05) or time limit of 3 days elapsed (Methods).

With the observation of co-segregation of ecDNAs, we next assessed the respective contributions of co-selection and co-segregation in shaping the patterns of ecDNA co-assortment using evolutionary modelling. Similar to previous work^12^, we implemented an individual-based, forward-time evolutionary framework to study ecDNA dynamics in a growing tumour population (Fig. 3a and Methods). This model is instantiated with a single founding cell carrying two distinct ecDNA species with the same copy number. Cells divide or die according to a ‘fitness’ function that determines their birth rate based on the presence of each ecDNA species. During cell division, ecDNA copies are inherited among daughter cells according to a ‘co-segregation’ parameter: a value of 0 indicates independent random segregation and a value of 1 indicates perfectly correlated segregation. By simulating 1 million cancer cells under fixed selection for two individual ecDNA species (Fig. 3b–e and Extended Data Fig. 8a,b), we found that (1) co-occurrence of ecDNA species is mainly driven by co-selection pressure acting over multiple generations with modest synergy from co-segregation (Fig. 3b,c); and (2) copy-number correlation of ecDNAs in cells is mainly driven by co-segregation alone, in which proportional copies of ecDNAs are inherited during cell division (Fig. 3d,e). Once a cancer cell population reaches high copy numbers, ecDNA co-occurrence becomes relatively stable (Extended Data Fig. 8b). We further validated these trends using an alternative model of ecDNA evolution (Methods and Extended Data Fig. 8c–e).

As co-selection and co-occurrence leave distinct signatures on the joint distributions of ecDNAs (Fig. 3f and Extended Data Fig. 8a), we sought to infer the levels of ecDNA co-selection and co-segregation based on experimentally observed ecDNA copy-number distributions in cells. Pairing our evolutionary model with ecDNA copy-number distributions obtained with scATAC-seq, we used approximate Bayesian computation (ABC)^47,48^ to infer posterior distributions for individual selection, co-selection and co-segregation of ecDNA species (Fig. 3g, Methods and Extended Data Fig. 8f–h). As validation, the inferred levels of co-segregation closely matched those experimentally observed in dividing cells using DNA-FISH (Fig. 2c,d and Fig. 3g and Extended Data Fig. 5d). This analysis inferred high levels of co-selection of ecDNA species relative to their individual selection in cancer cells (Extended Data Fig. 8g,h). Co-selection becomes less critical at higher initial copy numbers for our inference procedure (in effect widening the 95% credible interval) while the co-segregation parameter remains stable across copy numbers (Extended Data Fig. 8h), consistent with the idea that co-segregation of ecDNA species maintains their correlated distributions in cells even at high ecDNA abundance. Together, these results show that co-selection and co-segregation underpin the co-assortment of ecDNAs in cancer cell populations (Fig. 3h).

## An altruistic enhancer-only ecDNA

**Fig. 4:**
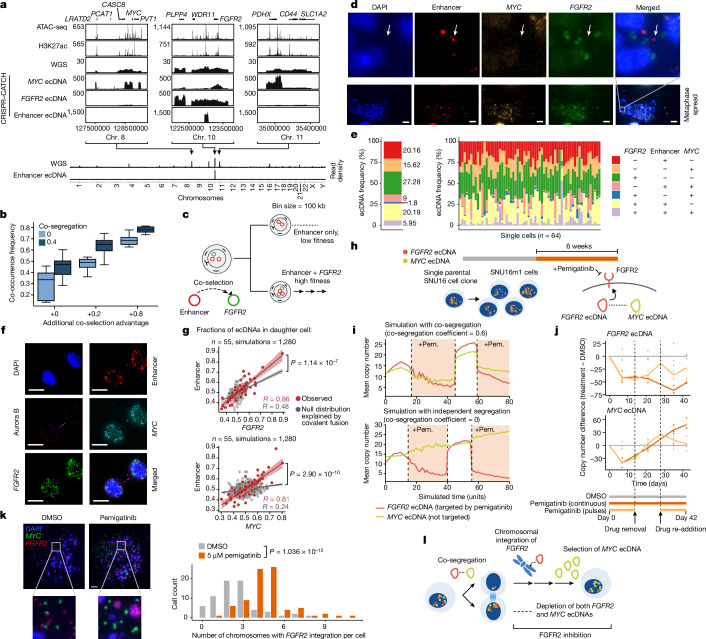
Specialization and therapeutic remodelling of ecDNA species. **a**, From top to bottom, ATAC-seq; H3K27ac ChIP–seq (chromatin immunoprecipitation followed by sequencing); WGS; CRISPR–CATCH sequencing of indicated ecDNAs in SNU16 cells; whole-genome read density plots of WGS and CRISPR–CATCH sequencing of enhancer ecDNA. **b**, Simulated co-occurrence of *FGFR2* and enhancer-only ecDNAs. Co-selection advantage is additive on cells carrying only *FGFR2* ecDNA. The box plots show the median (centre line), upper and lower quartiles (box limits), and 1.5× interquartile range (whiskers). *n* = 1,000,000 cells per 10 replicates per parameter set. **c**, Co-selection of enhancer ecDNA with *FGFR2* ecDNA. **d**,**e**, Representative metaphase DNA-FISH images of SNU16 cells targeting enhancer, *MYC* and *FGFR2* sequences (*n* = 64 cells) (**d**), and quantification of ecDNA frequencies (**e**). For **d**, scale bars, 10 µm. White arrows indicate an enhancer-only ecDNA species. **f**, Representative images of SNU16 mitotic cells identified by immunofluorescence for Aurora kinase B (*n* = 55 cell pairs). Individual ecDNAs were visualized using sequence-specific FISH probes. Scale bars, 10 µm. Enhancer DNA-FISH probe (hg19): chromosome 10: 123023934–123065872 (WI2-2856M1). **g**, Correlations of ecDNA species in one of each daughter cell pair compared to the simulated null distribution explained by covalent fusion (Pearson’s *R*; *P* values for the observed correlations compared with the null distributions were calculated using Fisher’s *z*-transformation and two-sided test). The error bands represent the 95% confidence intervals. **h**, FGFR2 inhibition with pemigatinib (pem.) in SNU16m1 cells. **i**, ecDNA copy numbers in simulated pemigatinib inhibition with or without co-segregation; drug decreases selection and co-selection values (−0.1 for cells carrying at least one copy of *FGFR2*). **j**, The copy-number difference of *FGFR2* and *MYC* ecDNAs in SNU16m1 cells with continuous or pulsed pemigatinib treatment compared with treatment with DMSO. **k**, Representative metaphase DNA-FISH images of SNU16m1 cells treated with pemigatinib (*n* = 81 cells) or DMSO (*n* = 66 cells) for 6 weeks (left). Right, quantification of chromosomes with *FGFR2* integration from metaphase spreads. Statistical analysis was performed using two-sided Wilcoxon rank-sum tests. Scale bars, 10 µm. **l**, Schematic of genomic changes under pemigatinib selection.

**Extended Data Fig. 9:**
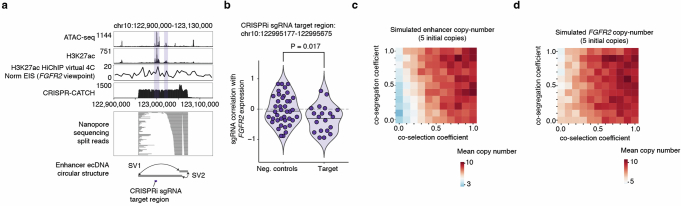
Structure and dynamics of the enhancer ecDNA. **(a)** From top to bottom: ATAC-seq, H3K27ac ChIP-seq, H3K27ac HiChIP contact with the *FGFR2* promoter, CRISPR-CATCH sequencing of enhancer-only ecDNA species in SNU16 cells, individual split reads in Nanopore sequencing supporting the circular enhancer-only ecDNA species, and structural variants (SV1 and SV2) that create a circular structure. Enhancer region targeted by CRISPR interference single-guide RNAs (sgRNAs) is marked at the bottom. SV1: precise inversion between chr10:122957191 and chr10:123051954; SV2: precise inversion between chr10:123058196 and chr10:123071737. **(b)** Correlations between individual sgRNAs and *FGFR2* expression after CRISPR interference followed by sorting of cells with various levels of *FGFR2* expression (data published and described in Hung et al.^4^). P-values determined by lower-tailed t-test compared to negative controls. Each dot represents an independent sgRNA (n = 40 negative control sgRNAs, n = 20 target sgRNAs). **(c-d)** Simulated copy number of enhancer-only ecDNA (**c**) and *FGFR2* ecDNA (**d**) under various settings of co-selection and co-segregation. Individual selection on the enhancer-only species was kept at 0.0, and individual selection on the *FGFR2* ecDNA was kept at 0.2. One million cells were simulated from a parent cell carrying 5 copies of both species. 10 replicates were simulated and the average value was reported. Norm EIS, normalized enhancer interaction signal.

We next assessed how co-selection and co-segregation contribute to the distributions of ecDNAs that do not themselves encode oncogenes but interact with other ecDNA molecules. We recently identified an ecDNA species in the parental SNU16 gastric cancer cell line that contains no oncogene-coding sequences but, instead, originated from a non-coding genomic region between *WDR11* and *FGFR2*. This region has accessible chromatin, is marked by histone H3 lysine 27 acetylation (H3K27ac) and contacts the *FGFR2* promoter, suggesting the presence of active enhancers^18^ (Fig. 4a and Extended Data Fig. 9a). At least one of these enhancer regions is required for oncogene activation on ecDNA, as evidenced by the reduced expression of *FGFR2* after targeting the enhancer region by CRISPR interference^4^ (Extended Data Fig. 9a,b). Long-read sequencing revealed that this enhancer ecDNA resulted from two inverted DNA segments joining together to create a circular molecule (Extended Data Fig. 9a). As intermolecular interactions of regulatory elements between different ecDNA molecules can drive oncogene expression^3,4^, the presence of amplified enhancer elements in the pool of ecDNA molecules may support enhancer–promoter interactions in *trans* and further upregulate oncogene expression—that is, an ‘altruistic’ ecDNA. An enhancer-only ecDNA may be especially sensitive to the co-occurrence of oncogene-coding ecDNAs in the same ecDNA hubs to exert its regulatory effect. Simulations under our model of ecDNA co-evolution suggested that co-segregation and co-selection synergize to maintain enhancer-only ecDNAs with oncogene-encoding ecDNAs in a majority of cancer cells (Fig. 4b,c) and that co-selection is particularly important to maintain enhancer-only ecDNAs (Extended Data Fig. 9c,d).

To quantify the frequency of enhancer-only ecDNA species, we performed metaphase DNA-FISH with separate, non-overlapping probes targeting the *MYC* and *FGFR2* coding sequences, as well as the enhancer sequence (Methods). This analysis showed that approximately 20% of ecDNA molecules in SNU16 cells contained this enhancer sequence without either oncogene (consistent with CRISPR–CATCH enrichment in the parental SNU16 line; Fig. 4a) and that the vast majority of individual cells (98%, 63 out of 64 cells examined) contained the enhancer-only ecDNA species (Fig. 4d,e). Analysis of pairs of daughter cells undergoing mitosis further showed co-segregation of the enhancer sequence with both *MYC* and *FGFR2* ecDNA molecules significantly above levels that can be explained by covalent linkages alone (*R* > 0.80, *P* < 1 × 10^−6^ for each comparison; Fig. 4f,g and Methods). These results support the theory that specialized ecDNAs without oncogenes can arise and be stably maintained by virtue of synergistic interaction with oncogene-carrying ecDNA.

## Pharmacological effects on ecDNA species

**Extended Data Fig. 10:**
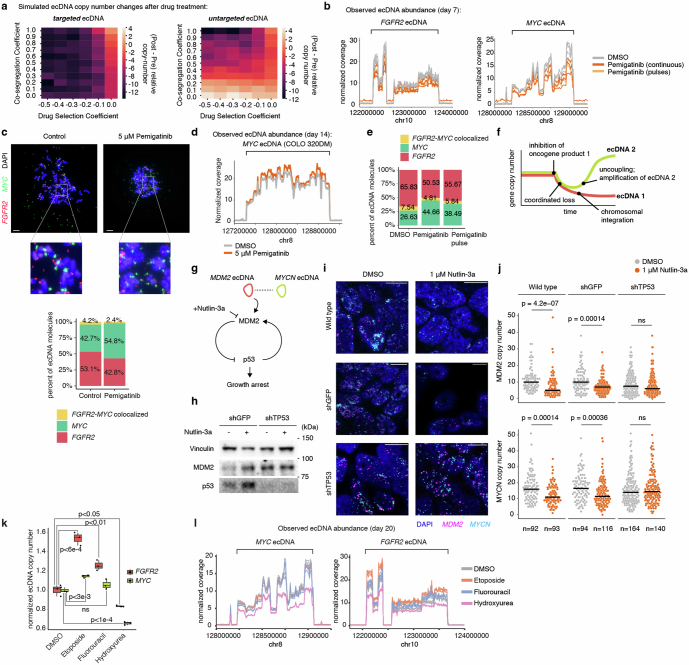
Characterization of pharmacological effects on ecDNA copy numbers. (**a**) Simulated changes in copy number after targeted treatment for the ecDNA directly or indirectly being targeted under various parameters of co-segregation and drug selection. 500,000 cells were simulated, and average values were reported across 10 replicates. **(b)** WGS coverage of *FGFR2* and *MYC* ecDNA genomic intervals after seven days of pemigatinib treatment at 5 µM compared to DMSO control. (**c)** Representative metaphase DNA FISH images showing distinct *FGFR2* and *MYC* ecDNA species in SNU16m1 cells after 20 days of treatment with 5 µM pemigatinib (top), and quantification of distinct and colocalized *FGFR2-MYC* DNA FISH signals (bottom). Control, n = 60 cells; pemigatinib, n = 58 cells. Scale bars, 10 µm. **(d)** WGS coverage of *MYC* ecDNA genomic interval in COLO 320DM cells after 14 days of treatment with 5 μM pemigatinib compared to DMSO control. **(e)** Quantification of distinct and colocalized *FGFR2-MYC* DNA FISH signals in metaphase DNA FISH images of *FGFR2* and *MYC* ecDNA species in SNU16m1 cells after 42 days of treatment with 5 µM pemigatinib or DMSO control. **(f)** Schematic of copy number changes of co-segregating ecDNA species under selective pressure. **(g)** Schematic of the inhibition of MDM2 as part of the p53 pathway. **(h)** Western blot analysis of TR14 cells with small hairpin RNA targeting either *GFP* (shGFP) or *TP53* (shTP53) with or without 1 μM nutlin-3a treatment. **(i)** Representative images of DNA FISH on interphase wild-type TR14 cells and cells with shGFP or shTP53 treated with 1 µM nutlin-3a or DMSO for 6 days (for DMSO treatments: wild-type, n = 92 cells; shGFP, n = 94 cells; shTP53, n = 164 cells; for nutlin-3a treatments: wild-type, n = 93 cells; shGFP, n = 116 cells; shTP53, n = 140 cells). Scale bars, 5 µm. **(j)** Copy numbers of *MDM2* and *MYCN* ecDNAs in wild-type TR14 cells, cells with shGFP or shTP53 after 6 days of 1 μM nutlin-3a or DMSO control treatment (p-values computed with a two-sided Wilcoxon rank-sums test). Each dot represents an individual cell (n represents number of cells in each condition) and horizontal lines show medians. **(k)** Copy-numbers of *MYC* and *FGFR2* in SNU16m1 cells after 20 days of treatment of DMSO control, 10 μM etoposide, 20 μM fluorouracil, or 100 μM Hyrdoxyurea. Biological replicates are shown as individual dots in the boxplots (n = 3 replicates for each sample). Statistical significance was assessed using a two-sided t-test (for *FGFR2*: p < 6e−4 for etoposide, p < 0.01 for fluorouracil, p < 0.05 for hydroxyurea; for *MYC*: p < 3e-3 for etoposide, n.s. for fluorouracil, p < 1e-4 for hydroxyurea). Boxplots show the quartiles of the distribution, centres indicate distribution median, and whiskers extend to 1.5x the interquartile range. **(l)** WGS coverage of *MYC* and *FGFR2* ecDNA genomic interval in SNU16m1 cells after 20 days of treatment with DMSO control, 10 μM etoposide, 20 µM fluorouracil, or 100 µM hydroxyurea. Source data

ecDNAs can drive rapid genome evolution in response to pharmacological treatment, including through modulation of copy number^29^ and generation of new ecDNAs containing resistance-promoting genes^11,18^. We hypothesized that co-segregation and co-selection of ecDNA species that interact in *trans* could lead to coupled copy-number dynamics in response to targeted drug treatment. To test this hypothesis, we performed drug treatment with pemigatinib, an FGFR2 inhibitor^49^, using the SNU16m1 gastric cancer monoclonal cell line (containing *MYC* and *FGFR2* ecDNAs that engage in intermolecular enhancer–promoter interactions^4^; Fig. 4h). Despite the clonal nature of the SNU16m1 cells, there is a high level of ecDNA copy-number heterogeneity among cells (5–300 copies of *MYC* ecDNA and 100–500 copies of *FGFR2* ecDNA in individual cells; Extended Data Fig. 2j). The *MYC* and *FGFR2* ecDNA species are correlated in copy number among these clonal cells (Extended Data Fig. 2j), consistent with the idea that a single-cell clone can establish heterogeneous yet correlated copy numbers of ecDNA species in progeny cells through asymmetric co-segregation during cell division. Pemigatinib was predicted to reduce the selective advantage of cells with amplified *FGFR2* expression, leading to loss of *FGFR2* ecDNAs in the cell population over time. When cells are treated with a drug that targets a single ecDNA species (such as pemigatinib targeting the gene product of *FGFR2* ecDNAs), our simulations predicted coordinated copy-number dynamics of co-existing ecDNAs only if they co-segregate (Fig. 4h,i, Methods and Extended Data Fig. 10a). Simulations further predicted that drug removal would allow steady recovery of the copy number of the targeted ecDNA species (Fig. 4i).

To test these predictions experimentally, we treated SNU16m1 cells with 5 μM pemigatinib over 6 weeks (Fig. 4h,j). As predicted by simulations of co-segregating ecDNAs, this targeted FGFR2 inhibition led to an initial coordinated depletion of both *FGFR2* and *MYC* ecDNAs (Fig. 4h,j and Extended Data Fig. 10b), supporting the idea that the two ecDNA species are coordinately inherited despite not being covalently linked (separate ecDNA species were validated by metaphase DNA-FISH after the first 3 weeks of drug treatment; Extended Data Fig. 10c,e). However, while cells that were continuously treated with pemigatinib maintained low *FGFR2* copy numbers, *MYC* ecDNA copy numbers recovered after week 3 and became further amplified, suggesting that *MYC* ecDNAs may eventually be selected in cells resistant to drug treatment (Fig. 4j (dark orange)). We further found that, while *MYC* had been selected on ecDNAs at high copy numbers, the remaining *FGFR2* copies increasingly integrated into chromosomes by week 6 (Fig. 4k). Importantly, while previous studies have reported that ecDNA can integrate into chromosomes^5,50,51^, our results suggest that its chromosomal integration can promote drug resistance by the evasion of co-inheritance (Fig. 4l and Extended Data Fig. 10f). A 2-week temporary removal of pemigatinib in the middle of the experiment resulted in recovery of *FGFR2* and *MYC* ecDNA copy numbers and re-established sensitivity to co-depletion of both ecDNA species once the drug was re-added, showing that the coordinated copy-number dynamics can be rapidly re-established within a few cell generations (Fig. 4j (light orange)). Finally, pemigatinib did not result in *MYC* ecDNA loss in the COLO 320DM colorectal cancer cell line, which does not contain *FGFR2* ecDNAs (Fig. 1c and Extended Data Fig. 10d), showing that the loss of *MYC* ecDNAs in SNU16m1 cells is specifically due to the coupling with *FGFR2* ecDNAs.

To further demonstrate the generality of these coordinated dynamics of ecDNA species under selective pressure, we treated the neuroblastoma TR14 cells with nutlin-3a, a targeted inhibitor of MDM2. MDM2 inhibition led to concomitant depletion of co-segregating *MDM2* and *MYCN* ecDNAs in a TP53-dependent manner, demonstrating molecular specificity of ecDNA co-depletion to MDM2 activity through the TP53 pathway (Methods and Extended Data Fig. 10g–j). Conversely, the coordinated depletion of ecDNAs under targeted inhibition cannot be explained by a general cytotoxic effect on rapidly dividing cells, as general cytotoxic drugs did not always reduce ecDNA contents (etoposide or fluorouracil; Extended Data Fig. 10k–l; low-dose hydroxyurea reduced ecDNA contents as reported previously^52,53^).

Together, these results demonstrate that pharmacological targeting of an oncogene carried by one ecDNA species can coordinately regulate co-existing ecDNA species, driven by both reduced selective advantage for a particular oncogene (for example, pemigatinib targeting *FGFR2*) and indirect effects on additional ecDNA species through physical co-segregation. However, resistance can emerge when ecDNA co-inheritance is uncoupled through chromosomal integration of the drug-targeted oncogene.

## Discussion

ecDNA amplifications in cancer are highly heterogeneous and dynamic, involving mixtures of DNA species that evolve and increase in complexity over time and in response to selective pressures such as drug treatments^31,54^. Through single-cell sequencing, imaging, evolutionary modelling and chemical perturbations across multiple cancer types, we have shown that diverse ecDNA species co-occur in cancer cells, that they co-segregate during mitosis, and that these evolutionary associations contribute to ecDNA specialization and response to targeted therapy. We have also shown that intermolecular interactions and active transcription promote co-segregation of ecDNA species. We provide evidence that ecDNA co-segregation is distinct from the damage-induced clustering of DNA fragments by the CIP2A–TOPBP1 complex^25,41^ (Extended Data Fig. 7), probably because the majority of ecDNAs lack double-stranded breaks (as shown by pulsed-field gel electrophoresis^18^), which are required for CIP2A recruitment^41^.

While individual ecDNAs are stochastically inherited during mitosis^12,14^, co-segregation and co-selection of distinct ecDNAs synergistically maintain a collective of cooperating ecDNAs across cell generations. This coordinated behaviour of ecDNA collectives presents implications for our understanding of cancer evolution and development of cancer therapies. First, co-selection of structurally diverse ecDNAs can lead to functional specialization (such as enhancer-only ecDNAs), suggesting that interactive modules of ecDNAs may exist, for example, within intermolecular ecDNA hubs^3,4,15^. Second, our pharmacological experiments show that therapeutic interventions targeting the gene product of an ecDNA species may impact co-existing ecDNAs and further underscore that co-segregation of ecDNA species gives rise to highly dynamic and complex behaviours under selective pressure. However, the eventual uncoupling of ecDNA species suggests that therapies naively exploiting co-segregation are not guaranteed to ‘cure’ tumour cells of ecDNA. Rather, acute targeted therapy can induce rapid, potentially therapeutically advantageous, genome remodelling as a consequence of ecDNA co-segregation. Third, our computational framework can assess ecDNA co-segregation and co-selection from single-cell genomic or imaging data, therefore offering opportunities to understand how ecDNAs co-evolve in tumours.

ecDNAs exhibit aggressive behaviour in cancer cells as they can rapidly shift in copy number and evolve novel gene regulatory relationships^4,12^. This accelerated evolution and ability to explore genetic and epigenetic space is challenged by its potentially transient nature—a winning combination of ecDNAs may not be present in the next daughter cell generation if they are randomly transmitted. ecDNA co-inheritance enables cancer cells to balance accelerated evolution with a measure of genetic and epigenetic memory across cell generations, increasing the probability that combinations of ecDNA species will be transmitted together to daughter cells (Fig. 3e). The consequence is a jackpot effect that supports cooperation among heterogeneous ecDNAs, enabling the co-amplification of multiple oncogenes and continued diversification of cancer genomes. Beyond cancer evolution, our general framework for coordinated asymmetric inheritance may be applicable to viral episomes, subcellular organelles or biomolecular condensates that control cell fates.

## Methods

### Cell culture

The TR14 neuroblastoma cell line was a gift from J. J. Molenaar (Princess Máxima Center for Pediatric Oncology). Cell line identity for the master stock was verified by STR genotyping (IDEXX BioResearch). The GBM39-KT cell line was derived from a patient with glioblastoma undergoing surgery at Mayo Clinic, Rochester, Minnesota as described previously^55^. Monoclonal spheroids were isolated from GBM39-KT cells by limiting dilution to generate GBM39-KT-D10. The CA718 cell line was derived from a patient with glioblastoma as described previously^5^ and was obtained from the University of California San Diego Moores Cancer Center. Parental SNU16, COLO 320DM, H716 and HCT116 cells were obtained from ATCC. The monoclonal SNU16m1 was a sub-line of the parental SNU16 cells generated from a single cell after lentiviral transduction and stable expression of dCas9-KRAB as we previously described^4^. SNU16 and SNU16m1 cells were maintained in Dulbecco’s modified Eagle’s medium/nutrient mixture F-12 (DMEM/F12 1:1; Gibco, 11320-082), 10% fetal bovine serum (FBS; Hyclone, SH30396.03) and 1% penicillin–streptomycin (Thermo Fisher Scientific, 15140-122). COLO 320DM cells were maintained in DMEM (Thermo Fisher Scientific, 11995073) supplemented with 10% FBS and 1% penicillin–streptomycin. GBM39-KT cells were maintained in DMEM/F12 1:1, B-27 supplement (Gibco, 17504044), 1% penicillin–streptomycin, GlutaMAX (Gibco, 35050061), human epidermal growth factor (EGF, 20 ng ml^−1^; Sigma-Aldrich, E9644), human fibroblast growth factor (FGF, 20 ng ml−1; Peprotech) and heparin (5 μg ml−1; Sigma-Aldrich, H3149-500KU). TR14 cells were grown in RPMI 1640 with 20% FBS and 1% penicillin–streptomycin. For the mitotic cell imaging experiments in Fig. 2, SNU16m1 cells were grown in RPMI 1640 with 10% FBS. H716 cells were grown in ATCC formulated RPMI 1640 (Gibco, A1049101) with 10% FBS and 1% penicillin–streptomycin–glutamine. COLO 320DM cells used for live-cell imaging, PC3 and HCT116 were cultured in DMEM (Corning, 10-013-CV) with 10% FBS and 1% penicillin–streptomycin–glutamine. All cells were cultured at 37 °C with 5% CO_2_. All cell lines tested negative for mycoplasma contamination.

### Chemicals

BRD4 bivalent degrader was a gift from M. M. Hassan and N. S. Gray, and was resuspended in DMSO as 10 mM stock^56^. Triptolide (Millipore, 645900) was resuspended with DMSO as 55 mM stocks and were used at a final concentration of 10 µM. Actinomycin D (Millipore Sigma, SBR00013) was used at a final concentration of 5 µg ml^−1^. DRB (Sigma-Aldrich, D1916) was resuspended with DMSO as 70 mM stocks and was used at a final concentration of 200 µg ml^−1^. ZL-12A was synthesized as reported previously^46^ and resuspended in DMSO as 20 mM stock, and was used at a final concentration of 50 µM for 3 h. In the pretreatment assay with triptolide, ZL-12A was added for 3 h, followed by a wash-off with 1× PBS and the addition of DMSO or triptolide (10 µM) for 3.5 h.

### Genetic knockout of *CIP2A*

*CIP2A-*knockout cells were created using the SNU16m1 cells as follows. We designed a guide RNA sequence targeting the protein-coding region of *CIP2A* using CHOPCHOP^57^ (https://chopchop.cbu.uib.no), as well as a non-targeting control sgRNA (guide sequences are provided in Supplementary Table 1). To deliver each guide with CRISPR–Cas9 into cells, we mixed purified *S. pyogenes* Cas9 nuclease (Alt-R S.p. Cas9 Nuclease V3; IDT, 1081058) with each single-guide RNA (sgRNA; diluted to 30 μM in 1× TE buffer; Synthego) at a 1:6 molar ratio in Neon Resuspension Buffer R (Thermo Fisher Scientific) and incubated it at room temperature for 10 min to form Cas9 ribonucleoprotein (RNP) complexes. SNU16m1 cells were collected and washed twice with 1× PBS before being resuspended in Buffer R with Cas9 RNPs for a final concentration of 300,000 cells per 10 μl Neon reaction with 0.71 μM Cas9 complexes. Transfection was performed using the Neon Transfection System (Thermo Fisher Scientific, MPK5000) according to the manufacturer’s protocol using 10 μl tips with the following parameters: 1,400 V, 20 m s^−1^, 2 pulses. Three Neon reactions per guide condition were combined, resulting in 900,000 cells for either the control or *CIP2A-*knockout genotype.

### WGS

WGS libraries were prepared by DNA tagmentation. We first transposed genomic DNA with Tn5 transposase produced as previously described^58^, in a 50 µl reaction with TD buffer^59^, 50 ng DNA and 1 µl transposase. The reaction was performed at 50 °C for 5 min, and transposed DNA was purified using the MinElute PCR Purification Kit (Qiagen, 28006). Libraries were generated by 5–7 rounds of PCR amplification using the NEBNext High-Fidelity 2× PCR Master Mix (NEB, M0541L), purified using SPRIselect reagent kit (Beckman Coulter, B23317) with double size selection (0.8× right, 1.2× left) and sequenced on the Illumina NextSeq 550 or the Illumina NovaSeq 6000 platform. Reads were trimmed of adapter content with Trimmomatic^60^ (v.0.39), aligned to the hg19 genome using BWA MEM^61^ (0.7.17-r1188) and PCR duplicates were removed using Picard’s MarkDuplicates (v.2.25.3). WGS data from bulk SNU16 cells were previously generated (SRR530826, Genome Research Foundation).

### Analysis of ecDNA sequences in TCGA patient tumours

We performed ecDNA detection based on bulk WGS data from TCGA using the AmpliconArchitect (AA) method for genomic focal amplification analysis. The outputs of this method were previously published^19^. In brief, this approach for detecting ecDNA uses three general steps which are wrapped into a workflow we call AmpliconSuite-pipeline (https://github.com/AmpliconSuite/AmpliconSuite-pipeline, v.1.1.1). First, given a BAM file, the analysis pipeline performs detection of seed regions where copy-number amplifications exist (CN > 4.5 and size between 10 kb and 10 Mb). Second, AA performs joint analysis of copy number and breakpoint detection in the focally amplified regions, forming a copy-number aware local genome graph. AA extracts paths representing genome structures and substructures from this graph that explains the changes in copy number. Last, a rule-based classification is performed using AmpliconClassifier (AC)^62^, based on the paths extracted by AA to predict the mode of focal amplification. This includes assessing structural variant types, segment copy numbers and the structure of the genome paths extracted by AA. Moreover, AC identifies ecDNA cycles based on criteria such as cyclic path length and copy number, providing a comprehensive classification system for amplicons on the basis of their structural characteristics. For example, if the changes in copy number are explained predominantly by one or more circular genome paths featuring a structural variant enclosing them with a head-to-tail circularization, this is consistent with an ecDNA mode of amplification, whereas a breakage-fusion-bridge genome structure contains multiple foldbacks and multiple genomic segments arranged in a palindrome. The complete classification criteria and description of the AC tool are available in the supplementary information of ref. ^62^.

We used AA (v.1.0) outputs from a previous study^19^, and classified focal amplifications types present in these outputs using AC (v.0.4.14) with the ‘--filter_similar’ flag set and otherwise the default settings. The ‘--filter_similar’ option removes probable false-positive focal amplification calls that contain far greater-than-expected levels of overlapping structural variants and shared genomic boundaries between ecDNAs of unrelated samples. In brief, AC scores the structural similarity of focal amplifications. These scores consider both genomic interval overlap and shared breakpoint junctions, with breakpoints deemed to be shared if their total distance is less than a specified threshold (default = 250 bp). Moreover, AC computes similarity scores for amplicons from unrelated origins, establishing a background null distribution for comparison. The tool uses a *β*-distribution model to fit the empirical null distribution, providing estimation of statistical significance of the similarity score. Out of 8,810 AA amplicons in the ref. ^19^ TCGA dataset, 45 candidate focal amplifications were removed by this filter.

To predict the distinct number of ecDNA species present in a sample, we used the genome intervals reported by AC for each focal amplification. AC determines the number of distinct, genomically non-overlapping ecDNA species present by clustering ecDNA genome intervals if those regions are connected by structural variants or the boundaries of the regions are within 500 kb. If intervals do not meet this criteria, AC predicts them as being unconnected and reports them as separate ecDNA species. AC uses a list of oncogenes that combines genes in the ONGene database (https://pubmed.ncbi.nlm.nih.gov/28162959/) and COSMIC (https://www.ncbi.nlm.nih.gov/pmc/articles/PMC6450507/).

### Paired scATAC-seq and scRNA-seq library generation

Single-cell paired RNA-seq and ATAC-seq libraries were generated on the 10x Chromium Single-Cell Multiome ATAC + Gene Expression platform according to the manufacturer’s protocol and sequenced on an Illumina NovaSeq 6000 system. Data for COLO 320DM were generated previously^4^ and published under Gene Expression Omnibus (GEO) accession GSE159986.

### Paired scATAC-seq and scRNA-seq analysis

A custom reference package for hg19 was created using cellranger-arc mkref (10x Genomics, v.1.0.0). The single-cell paired RNA-seq and ATAC-seq reads were aligned to the hg19 reference genome using cellranger-arc count (10x Genomics, v.1.0.0).

Subsequent analyses on RNA were performed using Seurat (v.3.2.3)^63^, and those on ATAC-seq were performed using ArchR (v.1.0.1)^64^. Cells with more than 200 unique RNA features, less than 20% mitochondrial RNA reads and less than 50,000 total RNA reads were retained for further analyses. Doublets were removed using ArchR. Raw RNA counts were log-normalized using Seurat’s NormalizeData function and scaled using the ScaleData function. Dimensionality reduction for the ATAC-seq data was performed using Iterative Latent Semantic Indexing (LSI) with the addIterativeLSI function in ArchR.

We next calculated amplicon copy numbers based on background ATAC-seq signals as we previously described and validated^4,32^. In brief, we determined read counts in large intervals across the genome using a sliding window of 3 Mb moving in 1 Mb increments across the reference genome. Genomic regions with known mapping artifacts were filtered out using the ENCODE hg19 blacklist. For each interval, insertions per bp were calculated and compared to 100 of its nearest neighbours with matched GC nucleotide content. The mean log_2_[fold change] was computed for each interval. On the basis of a diploid genome, copy numbers were calculated using the formula ${\rm{CN}}=2\times {2}^{{\log }_{2}[{\rm{FC}}]}$), where CN denotes copy number and FC denotes mean fold change compared with neighbouring intervals. To query the copy numbers of a gene, we obtained all genomic intervals that overlapped with the annotated gene sequence and computed the mean copy number of those intervals.

For analyses presented in Extended Data Fig. 5a–c, we inferred cell cycle stage from each cell’s RNA-seq data using the CellCycleScoring function in Seurat and the gene sets for S and G2M phases included in the Seurat package. Copy-number correlations were then evaluated for cells grouped by their inferred cell cycle phase: G1, S, or G2M.

### scCircle-seq analysis

TR14 scCircle-seq data were previously generated^65^ and deposited at the European Genome-Phenome Archive (EGA) under accession number EGAS00001007026. A detailed description of the single-cell extrachromosomal circular DNA and transcriptome sequencing (scEC&T-seq) protocol is available at Nature Protocol Exchange (10.21203/rs.3.pex-2180/v1)^66^. Single cells were sorted, separation of genomic DNA and mRNA was performed by G&T-seq^67^ and genomic DNA of single cells was subjected to exonuclease digestion and rolling-circle amplification as described previously^65^.

The processing of scCircle-seq reads is described in detail previously^65^. In brief, scCircle-seq sequencing reads were 3′ trimmed for quality using Trim Galore (v.0.6.4)^68^, and adapter sequences with reads shorter than 20 nucleotides were removed. The alignment of reads to the human reference assembly hg19 was performed using BWA MEM (v.0.7.15) with the default parameters^69^. PCR and optical duplicates were removed using Picard (v.2.16.0). Sequencing coverage across mitochondrial DNA was used as an internal control to evaluate circular DNA enrichment. Cells that exhibited less than 10 reads per bp sequence-read depth over mitochondrial DNA or less than 85% genomic bases captured in mitochondrial DNA were excluded from further analyses^65^.

Read counts from scCircle-seq BAM files were quantified in 1 kb bins across TR14 ecDNA regions (*MYNC*, *CDK4*, *MDM2*) as defined by ecDNA reconstruction analyses in TR14 bulk populations described previously^4^. To account for differences in sequencing depth among cells, read counts were normalized to library size.

### Analysis of copy-number correlations of amplified oncogenes in human tumour samples

Copy numbers computed for single cells using scATAC-seq as described above (see the ‘Paired scATAC-seq and scRNA-seq analysis’ section) were used to devise a statistical approach for predicting ecDNA. We reasoned that, due to the random segregation of individual ecDNA molecules, ecDNA focal amplifications would be characterized by not only elevated mean copy number but also inflated copy-number variance. Indeed, classifying amplifications with a mean copy number of ≥4 and variance/mean ratio of ≥2.5 specifically classified only known ecDNAs in validated cell lines (Extended Data Fig. 4a).

We applied this statistical approach to a curated dataset of 41 tumours (from triple-negative breast cancer (TNBC), high-grade serous ovarian cancer (HGSC) and glioblastoma) with publicly available scATAC-seq or scDNA-seq data^34–36^. For TNBC and HGSC tumours profiled with scDNA-seq data in ref. ^35^, we used the author-provided single-cell copy numbers available on Zenodo (10.5281/zenodo.6998936). Processed scATAC-seq data for glioblastoma samples were obtained from ref. ^34^ and ref. ^36^ (GEO accession number GSE163655), and copy numbers were computed as described above (see the ‘Paired scATAC-seq and scRNA-seq analysis’ section) in 3 Mb genomic windows. Putative ecDNAs were predicted using the decision rule determined from validated cell lines, and copy numbers were determined for oncogenes by averaging copy numbers of windows overlapping with the oncogene of interest. Copy-number correlations were computed across oncogenes, only considering cells where the oncogene was amplified with a copy-number ≥4.

### ecDNA isolation by CRISPR–CATCH

Molecular isolation of ecDNA by CRISPR–CATCH was performed as previously described^18^. In brief, molten 1% certified low-melting-point agarose (Bio-Rad, 1613112) in PBS was equilibrated to 45 °C. In total, 1 million cells were pelleted per condition, washed twice with cold 1× PBS, resuspended in 30 µl PBS and briefly heated to 37 °C. Then, 30 µl agarose solution was added to cells, mixed, transferred to a plug mould (Bio-Rad, 1703713) and incubated on ice for 10 min. Solid agarose plugs containing cells were ejected into 1.5 ml Eppendorf tubes, suspended in buffer SDE (1% SDS, 25 mM EDTA at pH 8.0) and placed onto a shaker for 10 min. The buffer was removed and buffer ES (1% *N*-laurolsarcosine sodium salt solution, 25 mM EDTA at pH 8.0, 50 µg ml^−1^ proteinase K) was added. Agarose plugs were incubated in buffer ES at 50 °C overnight. The next day, proteinase K was inactivated with 25 mM EDTA with 1 mM PMSF for 1 h at room temperature with shaking. Plugs were then treated with RNase A (1 mg ml^−1^) in 25 mM EDTA for 30 min at 37 °C and washed with 25 mM EDTA with a 5 min incubation. Plugs not directly used for ecDNA enrichment were stored in 25 mM EDTA at 4 °C.

To perform in vitro Cas9 digestion, agarose plugs containing DNA were washed three times with 1× NEBuffer 3.1 (New England BioLabs) with 5 min incubations. Next, DNA was digested in a reaction with 30 nM sgRNA (Synthego) and 30 nM spCas9 (New England BioLabs, M0386S) after pre-incubation of the reaction mix at room temperature for 10 min. Cas9 digestion was performed at 37 °C for 4 h, followed by overnight digestion with 3 µl proteinase K (20 mg ml^−1^) in a 200 µl reaction. The next day, proteinase K was inactivated with 1 mM PMSF for 1 h with shaking. The plugs were then washed with 0.5× TAE buffer three times with 5 min incubations. The plugs were loaded into a 1% certified low-melting-point agarose gel (Bio-Rad, 1613112) in 0.5× TAE buffer with ladders (CHEF DNA Size Marker, 0.2–2.2 Mb; *Saccharomyces cerevisiae* ladder, Bio-Rad, 1703605; CHEF DNA size marker, 1–3.1 Mb; *Hansenula wingei* ladder, Bio-Rad, 1703667) and pulsed-field gel electrophoresis was performed using the CHEF Mapper XA System (Bio-Rad) according to the manufacturer’s instructions and using the following settings: 0.5× TAE running buffer, 14 °C, two-state mode, run time duration of 16 h 39 min, initial switch time of 20.16 s, final switch time of 2 min 55.12 s, gradient of 6 V cm^−1^, included angle of 120° and linear ramping. The gel was stained with 3× Gelred (Biotium) with 0.1 M NaCl on a rocker for 30 min covered from light and imaged. The bands were then extracted and DNA was isolated from agarose blocks using beta-Agarase I (New England BioLabs, M0392L) according to the manufacturer’s instructions. All guide sequences are provided in Supplementary Table 1.

### Short-read sequencing of ecDNA isolated by CRISPR–CATCH

Sequencing of ecDNA isolated by CRISPR–CATCH was performed as previously described^18^. In brief, we transposed DNA with Tn5 transposase produced as previously described^58^ in a 50 µl reaction with TD buffer^59^, 10 ng DNA and 1 µl transposase. The reaction was performed at 50 °C for 5 min, and transposed DNA was purified using the MinElute PCR Purification Kit (Qiagen, 28006). The libraries were generated by 7–9 rounds of PCR amplification using NEBNext High-Fidelity 2× PCR Master Mix (NEB, M0541L), purified using SPRIselect reagent kit (Beckman Coulter, B23317) with double size selection (0.8× right, 1.2× left) and sequenced on the Illumina NextSeq 550 or the Illumina NovaSeq 6000 platform. Sequencing data were processed as described above for WGS. CRISPR–CATCH sequencing data for SNU16m1 (bands 30–34) and COLO 320DM (bands a–m) used in Extended Data Fig. 3 were generated previously^4^ and deposited at the NCBI Sequence Read Archive (SRA) under BioProject accession PRJNA670737; CRISPR–CATCH sequencing data for SNU16 (*MYC*, *FGFR2* and enhancer ecDNAs) used in Fig. 4 were generated previously^18^ and deposited at the NCBI SRA under BioProject accession PRJNA777710.

### Metaphase DNA-FISH

TR14 neuroblastoma cells were grown to 70% confluency in a 15 cm dish and treated with KaryoMAX Colcemid (Gibco) for 4 h. A mitotic shake off was performed and the medium of the cells was collected. The remaining cells were washed with PBS and treated with trypsin-EDTA 0.05% (Gibco) for 2 min. The cells were washed again with the collected medium and centrifuged at 300*g* for 10 min. The pellet was resuspended at 0.075 M KCl and left at 37 °C for 20 min. The sample was centrifuged at 300*g* for 5 min. The cell pellet was resuspended carefully in 10 ml Carnoy’s solution and centrifuged at 300*g* for 5 min. This wash step was repeated four times using 5 ml of Carnoy’s solution. The remaining pellet was resuspended in 400 µl of Carnoy’s solution. Then, 12 µl of the suspension was dropped on preheated slides from a height of approximately 15 cm. The slides were held over a heated water bath (55 °C) for 1 min. The slides were aged overnight at room temperature. Slides were prepared for staining according to the probe manufacturer’s protocol (DNA-FISH metaphase chromosome spreads, Arbor Biosciences). Before staining, the slides were first washed in PBS, followed by a wash in 65 °C SSCT (5 ml 20× SSC, 500 µl 10% Tween-20, and brought up to 50 ml with molecular-grade H_2_O) for 15 min. The slides were next washed twice for 2 min with room temperature SSCT. Dehydration of the slides was performed in 70% and 90% ethanol for 5 min each. After air-drying, the slides were transferred into 0.07 N NaOH for 3 min for chemical denaturation. After two washes for 5 min in SSCT, the dehydration step was repeated, and the slides were air-dried. The probes used for staining were designed to target the *MYCN*, *MDM2* and *CDK4* gene using myTags (Arbor), conjugated as following: CDK4-Alexa 488, MYCN-Atto 550, MDM2-Atto 633. Then, 10 µl of the hybridization buffer (in SSCT: 50 % formamide, 10% dextran sulphate, 40 ng µl^−1^ RNase A) was mixed with 1.5 µl of each resuspended probe. This mixture was headed to 70 °C for 5 min and stored on ice. Then, 14.5 µl of this mixture was added to the slide, which was covered by a cover glass and sealed with rubber cement. The slides were incubated in a hybridization chamber (Abbott Molecular) overnight at 37 °C. The next day, the rubber cement and cover glass were removed, and the sample was washed in prewarmed (37 °C) SSCT for 30 min. The slides were then washed at room temperature with 2× SSCT for 5 min each followed by a 5 min wash with PBS. The air-dried slide was stained with Hoechst (1: 4,000 for 2 min) and washed with PBS for another 5 min. After drying, the slides were mounted using ProLong Glass Antifade Mountant (Thermo Fisher Scientific) and sealed with a coverglass. Imaging of TR14 metaphase spreads was done on the Leica Stellaris 8 system (Advanced Light Microscopy Facility, Max-Delbrück Center for Molecular Medicine) using a ×63 oil objective with a ×2 zoom. Excitation was done using the 405 nm, 488 nm, 561 nm and 538 nm lasers and detection was done using two HyD S and one HyD X and HyD R detectors. 4× line averaging was applied to each channel.

For the GBM39-KT, GBM39-KT-D10, SNU16, SNU16m1, CA718 and H716 cell lines, cells were treated with KaryoMAX Colcemid (Gibco) at 100 ng ml^−1^ for 3 h, and single-cell suspensions were then collected by centrifugation and washed once in 1× PBS. The cells were treated with 0.75 M KCl hypotonic buffer for 20 min at 37 °C, and fixed with Carnoy’s fixative (3:1 methanol:glacial acetic acid) followed by three additional washes with the same fixative. The samples were then dropped onto humidified glass slides and air-dried. The glass slides were then briefly equilibrated in 2× SSC buffer, dehydrated in ascending ethanol concentrations of 70%, 85% and 100% for 2 min each. FISH probes (Empire Genomics) were diluted in hybridization buffer in 1:6 ratio and covered with a coverslip. The samples were denatured at 75 °C for 3 min and hybridized at 37 °C overnight in a humidified slide moat. The samples were washed with 0.4× SSC for 2 min, and 2× SSC 0.1% Tween-20 for another 2 min. The nuclei were stained with 4,6-diamidino-2-phenylindole (DAPI) (50 ng ml^−1^) diluted in 2× SSC for about a minute, and washed once briefly in double-distilled H_2_O. Air-dried samples were mounted with ProLong Diamond. Images were acquired on a Leica DMi8 widefield microscope using a 63× oil objective.

### Metaphase DNA-FISH image analysis

Colocalization analysis for two- and three-colour metaphase FISH described in Fig. 1 and Extended Data Fig. 2 was performed using Fiji (v.2.1.0/1.53c)^70^. Images were split into the individual FISH colours + DAPI channels, and the signal threshold was set manually to remove background fluorescence. Overlapping FISH signals were segmented using watershed segmentation. FISH signals were counted using particle analysis. *xy* coordinates of pixels containing FISH signals were saved along with image dimensions and coordinates of regions of interest (ROIs) as distinct particle identities (for example, distinct ecDNA molecules). Colocalization was then quantified in R. Each pixel containing FISH signal was assigned to the nearest overlapping ROI using *xy* coordinates. Unique ROIs in all colour channels were summarized such that ROIs in different channels that overlap with one another by one pixel or more in the same image were considered as colocalized.

Colocalization analysis for two-colour metaphase FISH data for ecDNAs in SNU16m1 cells described in Extended Data Fig. 10 was performed using Fiji (v.2.1.0/1.53c)^70^. Images were split into the two FISH colours + DAPI channels, and signal threshold set manually to remove background fluorescence. Overlapping FISH signals were segmented using watershed segmentation. Colocalization was quantified using the ImageJ-Colocalization Threshold program and individual and colocalized FISH signals were counted using particle analysis.

### Immunofluorescence staining and DNA-FISH in mitotic cells

For assessing mitotic segregation of ecDNA in GBM39-KT, GBM39KT-D10, TR14, SNU16m1, CA718 and H716 cells shown in Fig. 2 and Extended Data Figs. 5 and 7, asynchronous cells were grown on coverslips coated with either poly-l-lysine or poly-d-lysine (laminin for GBM39-KT and GBM39KT-D10). Cells were washed once with PBS and fixed with cold 4% paraformaldehyde (PFA) at room temperature for 10−15 min. The samples were permeabilized with 0.5% Triton X-100 in PBS for 10 min at room temperature and then washed with PBS. The samples were then blocked with 3% BSA in PBS 0.05% Triton X-100 for 30 min at room temperature. The samples were incubated in primary antibody (Aurora kinase B polyclonal antibody, 1:200 dilution, A300-431A, Thermo Fisher Scientific; BRD4 antibody, 1:200, ab245285, Abcam; RNA polymerase II CTD repeat YSPTSPS (phosphorylated Ser2) antibody (3E10), ab252855, Abcam; CIP2A antibody, 1:400 dilution, NBP2-48710, Novus Biologicals; all diluted in 3% BSA) for either 1 h at room temperature or overnight at 4 °C. The samples were washed three times in PBS 0.05% Triton X-100. The samples were incubated in fluorophore-conjugated secondary antibody (1:500 in 3% BSA) for 1 h at room temperature (with all of the subsequent steps in the dark) and then washed three times in PBS 0.05% Triton X-100. Cells were washed once with PBS and refixed with cold 4% PFA for 20 min at room temperature. The coverslips were then washed once in 1× PBS, and incubated with freshly prepared 0.7% Triton X-100 in 1× PBS with 0.1 M HCl for 10 min on ice, followed by acid denaturation of DNA strands with 1.9 M HCl for 30 min at room temperature. They were then dehydrated in ascending ethanol concentrations of 70%, 85% and 100% for approximately 2 min each. FISH probes (Empire Genomics) were diluted 1:4 in hybridization buffer (Empire Genomics) and added to the sample with the addition of a slide. The samples were denatured at 75 °C for 3 min and then hybridized at 37 °C overnight in a humid and dark chamber. The samples were then washed with 0.4× SSC then 2× SSC 0.1% Tween-20 (all washes lasting approximately 2 min). DAPI (100 ng ml^−1^) was applied to samples for 10 min. The samples were then washed again with 2× SSC 0.1% Tween-20 then 2× SSC. The samples were briefly washed in double-distilled H_2_O and mounted with ProLong Gold. The slides were sealed with nail polish. The samples were imaged either on a DeltaVision Elite Cell Imaging System (Applied Precision), on an Olympus widefield microscope (IX-71; Olympus) controlled by the SoftWoRx software v.6.5.2 (Applied Precision) and a ×60 objective lens with a CoolSNAP HQ2 camera (Photometrics), or on a Leica DMi8 widefield microscope using a ×63 oil objective lens. *z* stacks were acquired and used to generate maximum-intensity projections (ImageJ or LAS X) for downstream analysis. Images acquired on the Leica DMi8 were subjected to deconvolution using either small-volume computational clearing or large-volume computational clearing before making maximum-intensity projections.

For assessing mitotic segregation of oncogene and enhancer ecDNAs in SNU16 cells as shown in Fig. 4, cells were seeded onto fibronectin-coated 22 × 22 coverslips contained in a six-well culture plate at about 70% confluence. Then, 24 h after cell seeding, the cells were fixed with 4% PFA and permeabilized with 1× PBS containing 0.25% Triton X-100. The samples were blocked with 3% BSA-1× PBS for 1 h at room temperature, followed by primary antibody incubation (Aurora B kinase antibody; A300-431A; Thermo Fisher Scientific) (1:200 in 3% BSA) overnight at 4 °C. The sample was washed three times in 1× PBS followed by incubation with diluted an anti-rabbit Alexa Fluor 647 antibody (donkey anti-rabbit IgG (H+L) highly cross-adsorbed secondary antibody, Alexa Fluor 647, A31573, Invitrogen; 1:500 dilution in 3% BSA) for 1 h at room temperature. The sample is then washed three times in 1× PBS and fixed with 4% PFA for 20 min at room temperature. DNA-FISH was performed as described in the ‘Metaphase DNA-FISH’ section, with the conditions to heat denaturation changed to 80 °C for 20 min. Images were acquired on a Leica DMi8 widefield microscope using a ×63 oil objective, and each *z* plane was post-processed by small-volume computational clearing on LAS X before generating maximum-projection images.

### Mitotic cell imaging analysis

To quantify fractions of ecDNAs segregated to each daughter cell in pairs of dividing cells as shown in Fig. 2 and Extended Data Figs. 5 and 7, ecDNA pixel intensity was quantified from maximum intensity projections using ImageJ. ecDNA pixel intensity was measured using the ‘Integrated Density’ measurement from ImageJ. Before quantification, the background signal from FISH probes was removed uniformly for the entire image until all background signal from the daughter cell nuclei was removed. We further filtered out images with poor quality, those with overlapping nuclei that did not allow for accurate segmentation and those showing cells with unclear daughter cell pairings based on Aurora kinase B staining. To measure the fractions of ecDNAs segregated to daughter cells after inhibitor treatments, segmentation of daughter cells and measurement of DNA-FISH abundance was performed on maximum-intensity projections using AIVIA Software (Leica Microsystems). Individual machine-learning-based pixel classifiers were trained on the channels corresponding to the FISH probes of interest and DAPI to create confidence masks for FISH signal and nuclei, respectively. The confidence masks were used to create a recipe to segment individual FISH puncta and assign each punctum to a segmented daughter cell. The fractional inheritance of each ecDNA species was estimated by comparing the FISH area in the daughter cells of each corresponding pair. The abundances of proteins of interest (RNA Pol II pSer2, CIP2A and BRD4) were quantified using AIVIA software by measuring the pixel intensity values in the segmented nuclei.

To quantify the fractions of oncogene and enhancer ecDNAs segregated to daughter cells as shown in Fig. 4, the images were split into the different FISH colours + DAPI channels, and the signal threshold was set manually to remove background fluorescence using Fiji (v.2.1.0/1.53c)^70^. Overlapping FISH signals were segmented using watershed segmentation. All FISH colour channels except for DAPI were stacked and ROIs were drawn manually to identify the two daughter cells, after which the colour channels were split again and image pixel areas occupied by FISH signals were analysed using particle analysis. Fractions of ecDNAs in each daughter cell were estimated by fractions of FISH pixels in the given daughter cell.

### Intron RNA-FISH

Intron RNA-FISH was performed using Stellaris RNA FISH system (LGC Biosearch Technologies), with the manufacturer’s protocol for adherent cells. Intron RNA-FISH probe was designed against *MYC* intron 2 sequence (hg38) using the Stellaris Probe Designer tool (maximum number of probes = 48, oligo length = 20, minimum spacing length = 2), the final probe design for *MYC* intron 2 consists of 31 probes and was tagged with the Quasar 570 fluorophore. Images were acquired on the Leica DMi8 system using a ×63 oil objective to obtain *z* stack images, which underwent small-volume computational clearing before making maximum-intensity projections. For the RNase-A-treated negative control, cells were first fixed in 3.7% PFA, followed by digestion with RNase A (Thermo Fisher Scientific, EN0531) diluted to a final concentration of 200 µg ml^−1^ with 1× RNase-free PBS for 30 min at 37 °C. RNase A was washed off once with 1× RNase-free PBS before 70% ethanol permeabilization. Intron RNA-FISH staining was then continued as described in the manufacturer’s protocol for adherent cells.

### Live-cell imaging

The live-cell imaging cell line was engineered from COLO 320DM cells obtained from ATCC. In brief, the engineering involved the following key steps: (1) CRISPR-mediated knock-in of 96× TetO array into intergenic sites next to *MYC*, followed by puromycin selection for TetO-positive cells; (2) lentiviral infection of TetR-mNeonGreen, followed by sorting of mNeonGreen positive cells using flow cytometry to enable labelling of TetO inserted *MYC* locus; (3) monoclonal expansion of cells and evaluation by microscopy to select for clones that forms distinct mNeonGreen puncta with a good signal-to-noise ratio; (4) lentiviral infection of H2B-emiRFP670 was conducted to fluorescently label histone H2B protein, followed by sorting of emiRFP670 and mNeonGreen double-positive cells using flow cytometry. The final monoclonal cells were analysed using metaphase DNA-FISH to confirm good TetO labelling efficiency and that amplicons remained as ecDNA structures.

Cells were seeded onto poly-d-lysine coated 96-well glass-bottom plates 2 days before imaging. On the day of imaging, the medium was switched to FluoroBrite DMEM (Gibco, A1896701) supplemented with 10% FBS and 1× GlutaMax. Prolong live antifade reagent (Invitrogen, P36975) was used at 1:200 dilution to suppress photobleaching. Cells were imaged on a top stage incubator (Okolab) fitted onto a Leica DMi8 widefield microscope with a ×63 oil objective, with temperature (37 °C), humidity and CO_2_ (5%) controlled throughout the imaging experiment.

### Simulations of ecDNA segregation in pairs of daughter cells

To understand how co-segregation dynamics of ecDNAs in dividing cells may affect copy-number correlations in daughter cells, we simulated distributions of ecDNA copies among two daughter cells by random sampling using the sample function in R, for which the sample size is the total copy number of an ecDNA species multiplied by two (as a result of DNA replication). For a given fraction of one ecDNA species that co-segregates with the same fraction of another ecDNA species, the corresponding ecDNA copies were randomly distributed among two daughter cells but at the same ratio for both ecDNA species.

To compare observed ecDNA segregation with these simulations given a non-zero frequency of covalent fusions between two ecDNAs such as the low-level fusion events between different oncogene ecDNA species in various cell lines shown in Extended Data Fig. 2 or those between the enhancer and oncogene sequences shown in Fig. 4, the fraction of fused ecDNAs was treated as co-segregating ecDNAs in the simulations. To generate the expected distributions of enhancer and oncogene ecDNAs among daughter cells in Fig. 4, for each mitotic immunofluorescence and FISH image collected, the fractions of enhancer ecDNAs, oncogene ecDNAs and fused enhancer-oncogene ecDNAs were used to simulate 20 segregation events in which a fraction of ecDNAs corresponding to the fused molecules were perfectly co-segregated. The resulting copy-number correlations in simulated daughter cells represent the null distribution of ecDNAs explained by covalent fusion alone with no additional co-segregation between distinct ecDNA molecules.

### ATAC-seq

ATAC-seq data for SNU16 were previously published under GEO accession GSE159986 (ref. ^4^). Adapter-trimmed reads were aligned to the hg19 genome using Bowtie2 (v.2.1.0). Aligned reads were filtered for quality using samtools (v.1.9)^71^, duplicate fragments were removed using Picard’s MarkDuplicates (v.2.25.3) and peaks were called using MACS2 (v.2.2.7.1)^72^ with a *q*-value cut-off of 0.01 and with a no-shift model.

### ChIP–seq

ChIP–seq data for SNU16 were previously published under GEO accession GSE159986 (ref. ^4^). Paired-end reads were aligned to the hg19 genome using Bowtie2 (ref. ^73^) (v.2.3.4.1) with the --very-sensitive option after adapter trimming with Trimmomatic^60^ (v.0.39). Reads with MAPQ values of less than 10 were filtered using samtools (v.1.9) and PCR duplicates removed using Picard’s MarkDuplicates (v.2.20.3-SNAPSHOT). The ChIP–seq signal was converted to bigwig format for visualization using deepTools bamCoverage^74^ (v.3.3.1) with the following parameters: --bs 5 --smoothLength 105 --normalize Using CPM --scaleFactor 10.

### Evolutionary modelling of ecDNA copy-number framework

ecDNA copy number was simulated over growing cell populations using a forward-time simulation implemented in Cassiopeia^75^ (https://github.com/YosefLab/Cassiopeia). All simulations performed in this study were of two distinct ecDNA species in a growing cell population. Simulations were parameterized with (1) initial ecDNA copy numbers (initial copy number for ecDNA species *j* is denoted as ${k}_{{\rm{init}}}^{j}$); (ii) selection coefficients for cells carrying no ecDNA (*s*_−,−_), both ecDNAs (*s*_+,+_), or either ecDNA (*s*_−,+_ or *s*_+,−_; in this study, selection coefficients are treated as constant functions of the types of ecDNA species present in a cell); (3) a base birth rate (*λ*_base_ = 0.5); (4) and a co-segregation coefficient (*γ*). Optionally, a death rate can also be specified (*μ*).

Starting with the parent cell, a birth rate is defined based on the selection coefficient acting on the cell, $s\in \{{s}_{-,-},\,{s}_{-,+},\,{s}_{+,-},\,{s}_{+,+}\}$ as *λ*_1_ = *λ*_base_ × (1 + *s*). Then, a waiting time to a cell division event is drawn from an exponential distribution: *t*_b_ ~ exp(−*λ*_1_). When a death rate is also specified, a time to a death event is also drawn from an exponential distribution: *t*_d_ ~ exp(−*μ*). If *t*_b_ < *t*_d_, a cell division event is simulated and a new edge is added to the growing phylogeny with edge length *t*_b_; otherwise, the cell dies and the lineage is stopped. This process will continue until a user-defined stopping condition is specified—either a target cell number (for example, 1 million) or a target time limit.

During a cell division, ecDNAs are split among daughter cells (d_1_ and d_2_) according to the co-segregation coefficient, *γ*, and the ecDNA copy numbers of the parent cell *p*. In this study, this co-segregation is simulated using two different strategies to determine the effects of co-segregation (see the ‘Alternative model of ecDNA co-evolution’ section below). In the following description, let ${n}_{j}^{(i)}$ indicate the copy number of ecDNA species *j* in daughter cell *i* and let *N*_*j*_ indicate the copy number of ecDNA species *j* in the parent cell.

ecDNA species 1 is randomly split distributed to each daughter cell:

$${n}_{1}^{(1)} \sim {\rm{binomial}}(2{N}_{1},0.5)$$

$${n}_{1}^{(2)}=2{N}_{1}-{n}_{1}^{(1)}$$

Where binomial is the binomial probability distribution. To simulate co-segregation, for the second ecDNA species, copies are distributed to the daughter cells in proportion to the segregation coefficient *γ* and the copy number of the first ecDNA species in each daughter cell:

$${n}_{2}^{\left(1\right),\gamma }=\gamma \times 2{N}_{2}\times \frac{{n}_{1}^{\left(1\right)}}{2{N}_{1}}$$

$${n}_{2}^{\left(2\right),\gamma }=\gamma \times 2{N}_{2}\times \frac{{n}_{1}^{(2)}}{2{N}_{1}}$$

Then, the remainder of copies left over that were not passed with co-segregation are randomly distributed between daughter cells:

$${n}_{2}^{\left(1\right),r} \sim {\rm{binomial}}(2{N}_{2}-{n}_{2}^{\left(1\right),\gamma }-{n}_{2}^{(2),\gamma },0.5)$$

$${n}_{2}^{\left(2\right),r}=2{N}_{2}-{n}_{2}^{\left(1\right),r}-{n}_{2}^{\left(1\right),\gamma }-{n}_{2}^{\left(2\right),\gamma }$$

After this simulation, the output is a phylogeny *T* over *l* leaves (denoted by *L*) with ecDNA copy numbers ${k}_{j}^{i}$ for ecDNA species *j* in leaf *i*.

### Evolutionary modelling of ecDNA co-assortment trends

To simulate the trends of ecDNA copy-number dynamics, we used the evolutionary modelling framework described previously (see the ‘Evolutionary modelling of ecDNA copy-number framework’ section). We used the following fixed parameters: selection acting on individual ecDNA (*s*_−,+_,*s*_+,−_) of 0.2, selection acting on cells without ecDNA (*s*_−,__−_) of 0.0, a base birth rate (*λ*_base_) of 0.5, and initial ecDNA copy numbers for both species (${k}_{init}^{1}={k}_{init}^{2}$) of 5 in the parental cell. We varied co-selection (*s*_+,+_) and co-segregation (*γ*) between 0 and 1.0 and reported the fraction of cells reporting a copy-number of both ecDNAs above a threshold *m* (by default 1) and the Pearson correlation between ecDNA copy numbers in cells:

$$C=\frac{1}{|L|}\sum _{l\in L}I({k}_{l}^{1} > m,{k}_{l}^{2} > m)$$

$$\rho ={\rm{Pearson}}({{\bf{k}}}_{L}^{1},{{\bf{k}}}_{L}^{2})$$

Where ${k}_{l}^{i}$ is the copy number of ecDNA species *i* in leaf *l* and ${{\bf{k}}}_{L}^{i}$ is the vector of copy numbers of ecDNA species *i* across all cells.

For the results presented in Fig. 3b–e and Extended Data Fig. 8b, we simulated populations of 1 million cells and reported the average co-occurrence and correlation across 10 replicates.

### Inference of evolutionary parameters

ABC was used to determine evolutionary parameters in cell line data, specifically selection acting on individual ecDNAs (assumed to be equal between ecDNAs (*s*_−,+_,*s*_+,−_), the level of co-selection (*s*_+,+_), and the co-segregation coefficient (*γ*). In brief, ABC takes a parameter set $\theta$ from a prior or proposal distribution and simulates a dataset ${y}_{0}$ from this parameter set. If the simulated dataset matches the observed dataset within specified error tolerance ${\epsilon }$, then we accept the parameter set and update our posterior distribution $\pi (\theta |{y}_{0})$. In our case, we defined the priors over each parameter as follows:

$$\pi \left({s}_{-,+}),\pi ({s}_{+,-}\right) \sim {Unif}(0,1)$$

$$\pi \left({s}_{+,+}\right) \sim {Unif}(0,2)$$

$$\pi \left(\gamma \right) \sim {Unif}(0,1)$$

We used the evolutionary model presented above (see section titled “Evolutionary modelling of ecDNA copy-number framework”) to simulate datasets ${y}_{0}$ from the proposed parameter set *θ*, no death rate, a base birth rate *λ*_base_ = 0.5, and selection acting on cells without ecDNA *s*_−,−_ = 0.

Here our goal is to infer a posterior distribution over each evolutionary parameter given single-cell copy numbers observed from scATAC-seq data in a target cell line, denoted as *y*_obs_ (see the ‘Paired scATAC-seq and scRNA-seq analysis’ section above). To accomplish this, we chose to derive summary statistics describing the co-occurrence (proportion of cells carrying more than 2 copies of each gene amplified as ecDNA) and the Pearson correlation between the log-transformed copy numbers of ecDNAs for guiding our inference, denoted by *C*_obs_ and *ρ*_obs_, respectively. In each round of ABC, we simulated a dataset *y*_0_ of 500,000 cells and compared the summary statistics of this simulated dataset to the observed summary statistics using the following distance function:

$$D(\,{y}_{{\rm{obs}}},{y}_{0})=| {C}_{{\rm{obs}}}-{C}_{0}| +{\rm{| }}\,{\rho }_{{\rm{obs}}}-{\rho }_{0}\,{\rm{| }}$$

where *C*_0_ and *ρ*_0_ are the simulated co-occurrence and Pearson correlation, respectively. We used a tolerance of *ϵ* = 0.05 as our target error, and each ABC instance was run for up to 3 days. Each simulation was initialized with a parental cell with equal copy-number of initial ecDNA (${k}_{{\rm{init}}}^{1}={k}_{{\rm{init}}}^{2}$): in Fig. 3g this initial copy number was 5 although alternative initial conditions are explored in Extended Data Fig. 8f–h. We used the following summary statistics for each cell line: SNU16m1 (C_obs_ = 0.99, *ρ*_obs_ = 0.46); TR14 (C_obs_ = 0.96, *ρ*_obs_ = 0.26); GBM39-KT (C_obs_ = 0.67, *ρ*_obs_ = 0.36).

The specific implementation of this procedure was performed using a sequential Monte Carlo scheme (ABC-SMC) using the Python package pyabc (v.0.12.8). In brief, this approach performs sequential rounds of inference while computing a weight for the accepted parameters for each iteration. Further details of this procedure were reported previously^76–79^.

### Cell-level co-segregation model of ecDNA co-evolution

Previously, we introduced the co-segregation on the ecDNA element level inside of each cell, where an ecDNA element carrying one species is linked to another element with a probability defined as the co-segregation parameter. Here, we introduce an alternative model, in which ecDNA co-segregation is implemented at the cellular level. In each cell division, if a cell is chosen for proliferation, the number of ecDNA copies in that cell are doubled. We first have the randomly segregation of both ecDNA species following a binomial distribution separately, and then pair those with high copy numbers into the same daughter cells with a probability $\gamma \in [\mathrm{0,\; 1}]$. More precisely, *γ* describes the likelihood of extreme copy-number correlation, and 1 − *γ* describes the likelihood of extreme copy-number anticorrelation. If *γ* = 0.5, it is related to unbiased likelihood for both extreme scenarios, and it results in the modelling of standard random ecDNA proliferation without co-segregation.

In this model, the population growth is also modelled as a birth–death stochastic process and implemented by a standard Gillespie algorithm^12^. We start from a small initial population (a single cell or three cells) carrying a certain amount of ecDNA elements and recording the exact number of ecDNA copies for each cell through the simulation. Cells are chosen randomly but proportional to their fitness (1 + *s*) for proliferation, where *s* is the selection coefficient. Neutral proliferation is defined compared to fitness of cells without ecDNA (*s* = 0). If there is a fitness effect by carrying ecDNA, *s* > 0. For simplicity, in our models, we give a fixed selection coefficient for cells carrying either ecDNA and vary the selection coefficient for cells with both ecDNA to investigate the impact of co-selection in ecDNA co-evolution. For reporting, we discretize the population into three subpopulations, named pure, mix and free (no) ecDNA cells (Fig. 3g), which represent cells carrying just one type of ecDNA, both types or no ecDNA at all, respectively. For the results presented in Extended Data Fig. 8c–e, we simulated populations of 10,000 cells and reported summary statistics across 500 replicates.

### Evolutionary modelling of drug intervention

The evolutionary model described previously (see the ‘Evolutionary modelling of ecDNA copy-number framework’ section) was used to evaluate the effect of pemigatinib treatment on SNU16m1 cells. To do so, we modified the framework to allow for a burn-in period to simulate population growth without drug and then introduced a perturbation to selection coefficients at a defined timepoint.

Specifically, we allowed the cell population to grow to 5,000 cells under the following conditions: base birth rate (*λ*_base_) of 0.5, a death rate (*μ*) of 2.5, an initial ecDNA copy number for both species (${k}_{{\rm{init}}}^{1}={k}_{{\rm{init}}}^{2}$) of 10, and the following selection coefficients: *s*_−,−_ = 0; *s*_−,+_ = 0.15; *s*_+,−_ = 0.15; *s*_+,+_ = 0.8 (here, let cells carrying only *FGFR2* ecDNA be denoted by *s*_+,−_ and cells only carrying *MYC* ecDNA by *s*_−,+_).

For the experiments presented in Extended Data Fig. 10a in which we examine the dynamics of ecDNA copy-number after pemigatinib treatments cross a range of values, we simulated pemigatinib treatment by modulating the co-segregation level and selection pressures acting on cells after the 5,000 cell burn-in population was simulated. Specifically, we explored co-segregation parameters between 0 and 1, and selection pressure values ${s}_{+,+}={s}_{+,-}\in \{0,-\,0.1,\,-\,0.2,\,-\,0.3,\,-\,0.4,\,-\,0.5\}$. We then simulated 500,000 cells from the pre-treatment group of 5,000 cells while maintaining the same values for *γ*, *μ*, *λ*_birth_, *s*_−,−_ and *s*_−,+_.

For the pulsed pemigatinib treatment simulations presented in Fig. 4i, we used the same base birth rate, initial copy numbers, death rate and selection coefficients for the burn-in period of 5,000 cells. To simulate the first round of pemigatinib treatment, selection pressure values were set to *s*_+,+_ = s_+,−_ = −0.1 and 100,000 cells were simulated from the initial 5,000 cell pre-treatment group and 25,000 cells were sampled at random to continue for the drug holiday. During the drug holiday, 1.2 million cells were simulated according to initial selection parameters from the 25,000 cells sampled from the simulated drug treatment, with a modified base birth rate of 0.4 to model recovery times after drug treatment. After the drug holiday, 200,000 cells were sampled at random and a further drug treatment was simulated up until at least 110 time units according to the same selection parameters used in the first round of simulated pemigatinib treatment. For time-dependent functions of copy number reported in Fig. 4i, the mean copy numbers of both ecDNA species were computed in time bins of 5 up until the introduction of pemigatinib and bins of 1 afterwards.

### Evolutionary modelling of enhancer-only ecDNA

To examine the evolutionary principles of enhancer-only ecDNA, we used the previously described evolutionary model (see the ‘Evolutionary modelling of ecDNA copy-number framework’ section above) without death and fixed the following evolutionary parameters: *s*_+,−_ = 0.2, *s*_−,+_ = 0, *λ*_base_ = 0.5 and ${k}_{{\rm{init}}}^{1}={k}_{{\rm{init}}}^{2}=5$. We simulated ten replicates of 1-million cell populations a modulated co-selection coefficient *s*_+,+_ from [0, 1] and co-segregation coefficient *γ* from [0, 1]. In Fig. 4, we report the distribution of co-occurrence summary statistics *C* across these ten replicates.

### Nanopore sequencing of SNU16 genomic DNA

Genomic DNA from approximately 2 million SNU16 cells was extracted using the MagAttract HMW DNA Kit (Qiagen, 67563) and prepared for long-read sequencing using the Ligation Sequencing Kit V14 (Oxford Nanopore Technologies SQK-LSK114) according to the manufacturer’s instructions. Libraries were sequenced on a PromethION (Oxford Nanopore Technologies) using a 10.4.1 flow cell (Oxford Nanopore Technologies FLO-PRO114M).

Base calling from raw POD5 data was performed using Dorado (Oxford Nanopore Technologies, v.0.2.1+c70423e). Reads were aligned to hg19 using Winnowmap2 (ref. ^80^) (v.2.03) with the following parameters: -ax map-ont. Structural variants were called using Sniffles^81^ (v.2.0.7) using the following additional parameters: --output-rnames.

### Pemigatinib treatment of SNU16m1 and COLO 320DM cell lines

SNU16m1 and COLO 320DM cells were treated with 5 μM pemigatinib (Selleckchem, S0088), or with an equal volume of DMSO. Fresh pemigatinib was replenished approximately every 3–4 days. Approximately 1 million SNU16m1 cells were sampled from the DMSO condition, 300,000 cells from the pemigatinib-treated conditions at day 0, 7, 14, 21, 28, 35 and 42; genomic DNA was extracted using the DNeasy Blood & Tissue Kit (Qiagen, 69504), and subjected to WGS (see the ‘WGS’ section above). Approximately 2 million COLO 320DM cells were sampled at day 14, genomic DNA was extracted using the Quick DNA MiniPrep kit (Zymo Research; D0325) and subjected to WGS using the same procedure as above. Copy numbers for oncogene regions were computed using cnvkit (v.0.9.6.dev0)^82^.

### Chemotherapy treatment of SNU16m1 cell line

SNU16m1 cells were treated with 10 μM etoposide (Selleckchem, S1225), 20 μM fluorouracil (Selleckchem, S1209), 100 μM hydroxyura (Selleckchem, S1896), or equal volume DMSO control for 20 days. 2,300,000 SNU16m1 cells were plated in T-75 flasks for treatment with chemotherapeutic drugs and approximately 1,000,000 cells were seeded in T-25 flasks for treatment with DMSO control. Fresh chemotherapy drug was replenished at least every 7 days. On day 20 of the experiment, the remaining cells were collected and genomic DNA was extracted using the Quick DNA MiniPrep kit (Zymo Research, D0325) and subjected to WGS and analysis (see the ‘WGS’ section above). Copy numbers for oncogene regions were computed using cnvkit (v.0.9.10)^82^.

### Nutlin-3a treatment of TR14 cells and interphase DNA-FISH

A total of 175,000 TR-14 cells was seeded per well in 12-well plates. Cells were treated either with 0.1% DMSO or with 1 µl nutlin-3a (Sigma Aldrich, SML0580) for 6 days, without an additional wash-out period.

The samples were fixed using Carnoy’s solution (3:1 methanol:acetic acid). Fixed samples on coverslips or slides were briefly equilibrated in 2× SSC buffer. They were then dehydrated in ascending ethanol concentrations of 70%, 90% and 100% for approximately 2 min each. FISH probes were diluted in probe hybridization buffer and added to the sample with the addition of a coverslip or slide. The samples were denatured at 78 °C for 5 min and then hybridized at 37 °C overnight in a humid and dark chamber. The samples were washed twice in 0.4× SSC with 0.3% IGEPAL CA-630 for 2 min with agitation for the first 10–15 s. They were then washed once in 2× SSC with 0.1% IGEPAL CA-630 at room temperature for 2 min, again with agitation for the first 10–15 s. DAPI (100 ng ml^−1^) was applied to samples for 10 min. The samples were then washed again with 2× SSC and mounted with ProLong Antifade Mountant.

FISH and microscopy was performed in the same manner as TR14 was processed as described above (see the ‘Metaphase DNA-FISH image analysis’ section). Statistical significance was assessed using Wilcoxon rank-sum tests.

### *TP53* knockdown by shRNA

Lentiviruses were produced for *TP53* knockdown using short hairpin RNA (shRNA) targeting *TP53* (shTP53) or *GFP* (sgGFP) as a control. The shTP53 pLKO.1 puro plasmid was a gift from Y. Yu, Johannes Kepler Universität Linz. The shGFP pLKO.1 control plasmid was obtained from the RNAi Consortium, Broad Institute. HEK293T cells were transfected using TransIT-LT1 (Mirus) in a 2:1:1 ratio of lentiviral plasmid, psPAX2 and pMD2.G plasmids (Addgene) according to the TransIT-LT1 manufacturer’s protocol. Viral supernatant was collected 48 and 72 h after transfection, pooled, filtered and stored at −80 °C.

TR14 cells were transduced for 1 day in the presence of 8 µg ml^−1^ polybrene (Sigma-Aldrich). They were then grown in full medium for 1 day and selected with puromycin (2 μg ml^−1^) for 5–7 days.

### Western immunoblotting

A total of 800,000 cells was seeded in six-well plates and treated with either 0.1% DMSO or with the indicated concentration of nutlin-3a (Sigma Aldrich, SML0580) for 6 days, without an additional wash-out period. Whole-cell protein lysates were then prepared by lysing cells in radioimmunoprecipitation assay buffer supplemented with cOmplete Protease inhibitor (Roche) and PhosphStop (Roche). Protein concentrations were determined using the bicinchoninic acid assay (Thermo Fisher Scientific). Then, 30 µg of protein was denatured in Laemmli buffer at 95 °C for 10 min. The lysates were loaded onto 16% Tris-Glycine (Thermo Fisher Scientific) for gel electrophoresis. Proteins were transferred onto polyvinylidene fluoride membranes (Roche), blocked with 5% dry milk for 1 h and incubated with primary antibodies overnight at 4 °C, followed by secondary antibodies for 1 h at room temperature (MDM2 antibody (SMP14), Santa Cruz Biotechnology, sc-965, 1:200 dilution; p53 Antibody (DO-1), Santa Cruz Biotechnology, sc-126, 1:500 dilution; goat anti-mouse IgG (H+L) secondary antibody, HRP, Invitrogen, 31430, 1:2,000 dilution; vinculin monoclonal antibody (VLN01), Invitrogen, MA5-11690, 1:250 dilution). Chemiluminescent signal was detected using SuperSignal West Femto Maximum Sensitivity Substrate (Thermo Fisher Scientific) and the Fusion FX7 imaging system (Vilber Lourmat) using ImageLab. Unprocessed western blot images are provided as source data.

### Reporting summary

Further information on research design is available in the Nature Portfolio Reporting Summary linked to this article.

## Online content

Any methods, additional references, Nature Portfolio reporting summaries, source data, extended data, supplementary information, acknowledgements, peer review information; details of author contributions and competing interests; and statements of data and code availability are available at 10.1038/s41586-024-07861-8.

## Supplementary information

Supplementary Table 1 CRISPR guide RNA sequences.

Reporting Summary

## Source data

Source Data Extended Data Fig. 10

## Data Availability

Sequencing data generated for this study have been deposited at the NCBI SRA under BioProject accession PRJNA1127616. Source imaging data generated for this study have been deposited in the Stanford Digital Repository^83^ (10.25740/ff315yn8920). AmpliconClassifier output files containing ecDNA coordinates in TCGA samples are publicly available at figshare^84^ (10.6084/m9.figshare.24768555.v1). WGS data from bulk SNU16 cells were previously generated (SRR530826, Genome Research Foundation). Paired scATAC-seq and scRNA-seq data for COLO 320DM cells were generated previously and published at the GEO (GSE159986). TR14 scCircle-seq data were deposited in the European Genome-phenome Archive (EGA; EGAS00001007026). CRISPR–CATCH sequencing data integrated from previous studies were deposited in the SRA under BioProject accessions PRJNA670737 and PRJNA777710. ATAC-seq and ChIP–seq data for SNU16 were previously published at the GEO (GSE159986). Source data are provided with this paper.

## Code availability

The ecDNA evolutionary modelling framework used in this study is publicly available through Cassiopeia^75^ at GitHub (https://github.com/YosefLab/Cassiopeia). AmpliconClassifer is available at GitHub (https://github.com/jluebeck/AmpliconClassifier).
