# Supplementary material for: Coordinated inheritance of extrachromosomal DNAs in cancer cells
Source: Nature. 2024 Nov 6;635(8037):201–9. doi: 10.1038/s41586-024-07861-8 (PMC11541006; doi:10.1038/s41586-024-07861-8)
Supplement: Supplementary file 1 — CRISPR guide RNA sequences. [file 41586_2024_7861_MOESM1_ESM.pdf]

nature portfolio

https://doi.org/10.1038/s41586-024-07861-8

Supplementary information

Coordinated inheritance of extrachromosomal DNAs in cancer cells

In the format provided by the authors and unedited

Supplementary Tables

Supplementary Table 1.

**Guide RNA target sequence**	**Guide RNA information**
CCAGCAATCGTTAACCACTG	*MYC* guide 3
CTTCGGGGAGACAACGACGG	*MYC* guide 5
GTGATATTTGAACCGCCCTG	*MYC* guide 7
GTCTTGCTAGACTATACCAG	*MYC* guide 19
GGAGTCCATTGCACCGGCCG	*CIP2A* guide
GAACGACTAGTTAGGCGTGTA	Non-targeting control guide
