# Supplementary material for: Coordinated inheritance of extrachromosomal DNAs in cancer cells
Source: Nature. 2024 Nov 6;635(8037):201–9. doi: 10.1038/s41586-024-07861-8 (PMC11541006; doi:10.1038/s41586-024-07861-8)
Supplement: Supplementary file 2 — Reporting Summary [file 41586_2024_7861_MOESM2_ESM.pdf]

nature portfolio

Corresponding author(s): Howard Y. Chang & Paul S. Mischel

Last updated by author(s): Jun 22, 2024

Reporting Summary

Nature Portfolio wishes to improve the reproducibility of the work that we publish. This form provides structure for consistency and transparency in reporting. For further information on Nature Portfolio policies, see our Editorial Policies and the Editorial Policy Checklist.

Statistics

For all statistical analyses, confirm that the following items are present in the figure legend, table legend, main text, or Methods section.

n/a	Confirmed	
☐	☒	The exact sample size (*n*) for each experimental group/condition, given as a discrete number and unit of measurement
☐	☒	A statement on whether measurements were taken from distinct samples or whether the same sample was measured repeatedly
☐	☒	The statistical test(s) used AND whether they are one- or two-sided<br>*Only common tests should be described solely by name; describe more complex techniques in the Methods section.*
☒	☐	A description of all covariates tested
☐	☒	A description of any assumptions or corrections, such as tests of normality and adjustment for multiple comparisons
☐	☒	A full description of the statistical parameters including central tendency (e.g. means) or other basic estimates (e.g. regression coefficient) AND variation (e.g. standard deviation) or associated estimates of uncertainty (e.g. confidence intervals)
☐	☒	For null hypothesis testing, the test statistic (e.g. *F*, *t*, *r*) with confidence intervals, effect sizes, degrees of freedom and *P* value noted<br>*Give P values as exact values whenever suitable.*
☐	☒	For Bayesian analysis, information on the choice of priors and Markov chain Monte Carlo settings
☒	☐	For hierarchical and complex designs, identification of the appropriate level for tests and full reporting of outcomes
☐	☒	Estimates of effect sizes (e.g. Cohen's *d*, Pearson's *r*), indicating how they were calculated

Our web collection on statistics for biologists contains articles on many of the points above.

Software and code

Policy information about availability of computer code

Data collection

Paired single-cell ATAC-seq and RNA-seq analysis
A custom reference package for hg19 was created using cellranger-arc mkref (10x Genomics, version 1.0.0). The single-cell paired RNA and ATAC-seq reads were aligned to the hg19 reference genome using cellranger-arc count (10x Genomics, version 1.0.0).
Subsequent analyses on RNA were performed using Seurat (version 3.2.3), and those on ATAC-seq were performed using ArchR (version 1.0.1). Cells with more than 200 unique RNA features, less than 20% mitochondrial RNA reads, less than 50,000 total RNA reads were retained for further analyses. Doublets were removed using ArchR (version 1.0.1). Raw RNA counts were log-normalized using Seurat's NormalizeData function and scaled using the ScaleData function. Dimensionality reduction for the ATAC-seq data were performed using Iterative Latent Semantic Indexing (LSI) with the addIterativeLSI function in ArchR. We then calculated amplicon copy numbers by determining read counts in large intervals across the genome using a sliding window of three megabases moving in one-megabase increments across the reference genome. Genomic regions with known mapping artifacts were filtered out using the ENCODE hg19 blacklist. For each interval, insertions per base pair were calculated and compared to 100 of its nearest neighbors with matched GC nucleotide content. Mean log2(fold change) was computed for each interval. Based on a diploid genome, copy numbers were calculated using the formula CN = 2 * [2 ^ log2(FC)], where CN denotes copy number and FC denotes mean fold change compared to neighboring intervals. To query the copy numbers of a gene, we obtained all genomic intervals that overlapped with the annotated gene sequence and and computed the mean copy number of those intervals.

ChIP-seq
ChIP-seq data for SNU16 were previously published under GEO accession GSE1599869. Paired-end reads were aligned to the hg19 genome using Bowtie2 (version 2.3.4.1) with the --very-sensitive option following adapter trimming with Trimmomatic (version 0.39). Reads with MAPQ values less than 10 were filtered using samtools (version 1.9) and PCR duplicates removed using Picard's MarkDuplicates (version 2.20.3-SNAPSHOT). ChIP-seq signal was converted to bigwig format for visualization using deepTools bamCoverage (version 3.3.1) with the

following parameters: --bs 5 --smoothLength 105 --normalizeUsing CPM --scaleFactor 10.

Nanopore sequencing of SNU16 genomic DNA
Basecalling from raw POD5 data was performed using Dorado (Oxford Nanopore Technologies, version 0.2.1+c70423e). Reads were aligned using Winnowmap2 (version 2.03) with the following parameters: -ax map-ont. Structural variants were called using Sniffles (version 2.0.7) using the following additional parameters: --output-rnames.

Whole Genome Sequencing
Reads were trimmed of adapter content with Trimmomatic (version 0.39), aligned to the hg19 genome using BWA MEM (0.7.17-r1188), and PCR duplicates removed using Picard's MarkDuplicates (version 2.25.3). WGS data from bulk SNU16 cells were previously generated (SRR530826, Genome Research Foundation).

Data analysis

Analysis of ecDNA sequences in TCGA patient tumors:
We performed ecDNA detection based on bulk WGS data from TCGA using the AmpliconArchitect (AA) method for genomic focal amplification analysis. The outputs of this method were previously published. In brief, this approach for detecting ecDNA uses three general steps which are wrapped into a workflow we call AmpliconSuite-pipeline (https://github.com/AmpliconSuite/AmpliconSuite-pipeline, version 1.1.1). First, given a BAM file, the analysis pipeline performs detection of seed regions where copy number amplifications exist (CN > 4.5 and size between 10 kbp and 10 Mbp). Second, AA performs joint analysis of copy number and breakpoint detection in the focally amplified regions, forming a copy-number aware local genome graph. AA extracts paths representing genome structures and substructures from this graph that explains the changes in copy number. Last, a rule-based classification is performed using AmpliconClassifier (AC), based on the paths extracted by AA to predict the mode of focal amplification. This includes assessing SV types, segment copy numbers and the structure of the genome paths extracted by AA. Additionally, AC identifies ecDNA cycles based on criteria such as cyclic path length and copy number, providing a comprehensive classification system for amplicons based on their structural characteristics. For instance, if the changes in copy number are explained predominantly by one or more circular genome paths featuring a structural variant enclosing them with a head-to-tail circularization, this is consistent with an ecDNA mode of amplification, whereas a breakage-fusion-bridge (BFB) genome structure contains multiple foldbacks and multiple genomic segments arranged in a palindrome. The complete classification criteria and description of the AC tool are available from the Supplementary Information document in Luebeck et al., 2023.

We utilized AmpliconArchitect (v1.0) outputs from Kim et al. 2020, and classified focal amplifications types present in these outputs using AmpliconClassifier (v0.4.14) with the "—filter_similar" flag set and otherwise default settings. The "—filter_similar" option removes likely false positive focal amplification calls which contain far greater-than-expected levels of overlapping SVs and shared genomic boundaries between ecDNAs of unrelated samples. In brief, AC scores the structural similarity of focal amplifications. These scores consider both genomic interval overlap and shared breakpoint junctions, with breakpoints deemed shared if their total distance is less than a specified threshold (default = 250bp). Additionally, AC computes similarity scores for amplicons from unrelated origins, establishing a background null distribution for comparison. The tool employs a beta distribution model to fit the empirical null distribution, providing estimation of statistical significance of the similarity score. Of 8810 AA amplicons in the Kim et al. TCGA dataset, 45 candidate focal amplifications were removed by this filter.
To predict the distinct number of ecDNA species present in a sample, we utilized the genome intervals reported by AC for each focal amplification. AC determines the number of distinct, genomically non-overlapping ecDNA species present by clustering ecDNA genome intervals if those regions are connected by structural variants or the boundaries of the regions are within 500kbp. If intervals do not meet this criteria, AC predicts them as being unconnected and reports them as separate ecDNA species. AC uses a list of oncogenes which combines genes in the the ONGene database (https://pubmed.ncbi.nlm.nih.gov/28162959/) and COSMIC (https://www.ncbi.nlm.nih.gov/pmc/articles/PMC6450507/).

Single-cell Circle-seq analyses
The processing of scCircle-seq reads is described in detail in Chamorro González et al. 2023. In short, scCircle-seq sequencing reads were 3′ trimmed for quality using Trim Galore (version 0.6.4), and adapter sequences with reads shorter than 20 nucleotides were removed. The alignment of reads to the human reference assembly hg19 was performed using BWA MEM (version 0.7.15) with default parameters. PCR and optical duplicates were removed using Picard (version 2.16.0). Sequencing coverage across mitochondrial DNA was used as an internal control to evaluate circular DNA enrichment. Cells that exhibited less than 10 reads per base pair sequence-read depth over mitochondrial DNA or less than 85% genomic bases captured in mitochondrial DNA were excluded from further analyses.
Read counts from scCircle-seq bam files were quantified in 1kb bins across TR14 ecDNA regions (MYNC, CDK4, MDM2) as defined by ecDNA reconstruction analyses in TR14 bulk populations described in Hung et al. 2021. To account for differences in sequencing depth among cells, read counts were normalized to library size.

Analysis of copy-number correlations of amplified oncogenes in human tumor samples
Copy numbers computed for single cells using scATAC-seq as described above (see section "Paired single-cell ATAC-seq and RNA-seq analysis") were utilized to devise a statistical approach for predicting ecDNA. We reasoned that due to the random segregation of individual ecDNA molecules, ecDNA focal amplifications would be characterized by not only elevated mean copy number but also inflated copy-number variance. Indeed, classifying amplifications with a mean copy-number >= 4 and variance/mean ratio >= 2.5 specifically classified only known ecDNAs in validated cell lines (Extended Data Figure 4a).
We applied this statistical approach to a curated dataset of 41 tumors (from triple-negative breast cancer [TNBC], high-grade serous ovarian cancer [HGSC], and glioblastoma) with publicly-available scATAC or scDNA-seq data. For TNBC and HGSC tumors profiled with sc-DNAseq in Funnell et al., we used the author-provided single-cell copy-numbers available on Zenodo (10.5281/zenodo.6998936). Processed scATAC-seq data for glioblastoma samples were obtained from Guilhamon et al. and Nikolic et al. (GEO accession number GSE163655), and copy numbers were computed as described above (see section "Paired single-cell ATAC-seq and RNA-seq analysis") in 3-Mb genomic windows. Putative ecDNAs were predicted using the decision rule determined from validated cell lines, and copy numbers were determined for oncogenes by averaging copy numbers of windows overlapping with the oncogene of interest. Copy-number correlations were computed across oncogenes, only considering cells where the oncogene was amplified with a copy-number >= 4.

Metaphase DNA FISH image analysis
Colocalization analysis for two- and three-color metaphase FISH described in Figure 1 and Extended Data Figure 1 was performed using Fiji (version 2.1.0/1.53c). Images were split into the individual FISH colors + DAPI channels, and signal threshold set manually to remove background fluorescence. Overlapping FISH signals were segmented using watershed segmentation. FISH signals were counted using particle analysis. XY coordinates of pixels containing FISH signals were saved along with image dimensions and coordinates of regions of interest (ROIs) as distinct particle identities (e.g. distinct ecDNA molecules). Colocalization was then quantified in R. Each pixel containing FISH signal was assigned to the nearest overlapping ROI using XY coordinates. Unique ROIs in all color channels were summarized such that ROIs in different

channels that overlap with one another by one pixel or more in the same image were considered as colocalized.

Colocalization analysis for two-color metaphase FISH data for ecDNAs in SNU16 cells described in Extended Data Figure 8c was performed using Fiji (version 2.1.0/1.53c). Images were split into the two FISH colors + DAPI channels, and signal threshold set manually to remove background fluorescence. Overlapping FISH signals were segmented using watershed segmentation. Colocalization was quantified using the ImageJ-Colocalization Threshold program and individual and colocalized FISH signals were counted using particle analysis.

Mitotic cell imaging analysis
To quantify fractions of ecDNAs segregated to each daughter cell in pairs of dividing cells as shown in Figure 2, ecDNA pixel intensity were quantified from maximum intensity projections using the ImageJ software. ecDNA pixel intensity was measured using the "Integrated Density" measurement from ImageJ. Prior to quantification, background signal from FISH probes was removed uniformly for the entire image until all background signal from the daughter cell nuclei was removed.
To measure fractions of oncogene and enhancer ecDNAs segregated to daughter cells in dividing cells as shown in Figure 4, Images were split into the different FISH colors + DAPI channels, and signal threshold set manually to remove background fluorescence using Fiji (version 2.1.0/1.53c). Overlapping FISH signals were segmented using watershed segmentation. All FISH color channels except DAPI were stacked and ROIs were drawn manually to identify the two daughter cells, after which the color channels were split again and image pixel areas occupied by FISH signals were analyzed using particle analysis. Fractions of ecDNAs in each daughter cell were estimated by fractions of FISH pixels in the given daughter cell.

For manuscripts utilizing custom algorithms or software that are central to the research but not yet described in published literature, software must be made available to editors and reviewers. We strongly encourage code deposition in a community repository (e.g. GitHub). See the Nature Portfolio guidelines for submitting code & software for further information.

Data

Policy information about availability of data

All manuscripts must include a data availability statement. This statement should provide the following information, where applicable:

- Accession codes, unique identifiers, or web links for publicly available datasets
- A description of any restrictions on data availability
- For clinical datasets or third party data, please ensure that the statement adheres to our policy

Sequencing data generated for this study are deposited in the NCBI Sequence Read Archive (SRA) under BioProject accession PRJNA1127616. AmpliconClassifier output files containing ecDNA coordinates in TCGA samples are made publicly available on FigShare (https://doi.org/10.6084/m9.figshare.24768555.v1). WGS data from bulk SNU16 cells were previously generated (SRR530826, Genome Research Foundation). Paired single-cell ATAC-seq and RNA-seq data for COLO 320DM were generated previously and published under GEO accession GSE159986. TR14 scCircle-seq data were deposited in the European Genome-phenome Archive (EGA) under the accession number: EGAS00001007026. CRISPR-CATCH sequencing data integrated from previous studies were deposited in the SRA under BioProject accessions PRJNA670737 and PRJNA777710. ATAC-seq and ChIP-seq data for SNU16 were previously published under GEO accession GSE159986. Source Data are provided with the paper.

Research involving human participants, their data, or biological material

Policy information about studies with human participants or human data. See also policy information about sex, gender (identity/presentation), and sexual orientation and race, ethnicity and racism.

Reporting on sex and gender	N/A
Reporting on race, ethnicity, or other socially relevant groupings	N/A
Population characteristics	N/A
Recruitment	N/A
Ethics oversight	N/A

Note that full information on the approval of the study protocol must also be provided in the manuscript.

Field-specific reporting

Please select the one below that is the best fit for your research. If you are not sure, read the appropriate sections before making your selection.

☒ Life sciences ☐ Behavioural & social sciences ☐ Ecological, evolutionary & environmental sciences

For a reference copy of the document with all sections, see nature.com/documents/nr-reporting-summary-flat.pdf

Life sciences study design

All studies must disclose on these points even when the disclosure is negative.

Sample size	No sample size calculation was performed. In the case of single-cell sequencing, we aimed to sequence thousands of cells per cell line, as is supported by a single reaction. For sequencing studies, we sequenced DNA from at least 1,000,000 cells which captures much of the genetic heterogeneity in a cancer population. Imaging quantifications included 9 or more cells for assessing differences between treatments to capture cell-to-cell variability.
Data exclusions	Single cell analysis:<br>Cells with more than 200 unique RNA features, less than 20% mitochondrial RNA reads, less than 50,000 total RNA reads were retained for further analyses. Doublets were removed using ArchR.
Replication	Computational experiments were replicated at least 10 times to determine confidence intervals around estimates. Cell line experiments were replicated at multiple doses or in orthogonal cell lines to confirm biological effect. Drug treatment experiments were performed in triplicates. All replication efforts were successful.
Randomization	All experiments used cultured cell lines. As we were able to directly test the effects of genetic and therapeutic perturbations on cell viability (in biological replicates), and investigators were not blinded to allocation during experiments and outcome assessment, randomization was not relevant to this study.
Blinding	All data were collected using instruments without bias. Because these data were generated using objective quantifications, researchers assessing results were not blinded for the experimental design. Blinding is not relevant to this study.

Reporting for specific materials, systems and methods

We require information from authors about some types of materials, experimental systems and methods used in many studies. Here, indicate whether each material, system or method listed is relevant to your study. If you are not sure if a list item applies to your research, read the appropriate section before selecting a response.

Materials & experimental systems

n/a	Involved in the study	
☐	☒	Antibodies
☐	☒	Eukaryotic cell lines
☒	☐	Palaeontology and archaeology
☒	☐	Animals and other organisms
☒	☐	Clinical data
☒	☐	Dual use research of concern
☒	☐	Plants

Methods

n/a	Involved in the study	
☒	☐	ChIP-seq
☒	☐	Flow cytometry
☒	☐	MRI-based neuroimaging

Antibodies

Antibodies used	Aurora B Polyclonal Antibody, 1:200 dilution, catalog no. A300-431A, ThermoFisher Scientific; BRD4 antibody, 1:200, catalog no. ab245285, Abcam; RNA polymerase II CTD repeat YSPTSPS (phospho S2) antibody [3E10], catalog no. ab252855, Abcam; CIP2A Antibody, 1:400 dilution, catalog no. NBP2-48710, Novus Biologicals; anti-rabbit Alexa Fluor 647 antibody (Donkey anti-Rabbit IgG (H+L) Highly Cross-Adsorbed Secondary Antibody, Alexa Fluor™ 647, catalog no. A31573, Invitrogen; 1:500 dilution in 3% BSA); MDM2 Antibody (SMP14), Santa Cruz Biotechnology, catalog no. sc-965, 1:200 dilution; P53 Antibody (DO-1), Santa Cruz Biotechnology, catalog no. sc-126, 1:500 dilution; Goat anti-Mouse IgG (H+L) Secondary Antibody, HRP, Invitrogen, catalog no. 31430, 1:2000 dilution; Vinculin Monoclonal Antibody (VLN01), Invitrogen, catalog no. MA5-11690, 1:250 dilution
Validation	All antibodies were validated by the manufacturers.<br><br>All antibodies are validated to react with corresponding human antigens. Citation data are acquired from CiteAb database:<br><br>Aurora B Polyclonal Antibody (A300-431A), 15 citations<br>BRD4 antibody (ab245285), 0 citations, validated by manufacturer for western blot, immunohistochemistry and immunoprecipitation<br>RNA polymerase II CTD repeat YSPTSPS (phospho S2) antibody (ab252855), 6 citations<br>CIP2A antibody (NBP2-48710), 1 citation<br>Donkey anti-Rabbit IgG (H+L) Highly Cross-Adsorbed Secondary Antibody, Alexa Fluor™ 647, 2353 Citations<br>MDM2 Antibody (SMP14), Santa Cruz Biotechnology, 1001 citations<br>P53 Antibody (DO-1), Santa Cruz Biotechnology, 5522 citations<br>Goat anti-Mouse IgG (H+L) Secondary Antibody, HRP, Invitrogen, 2106 citations<br>Vinculin Monoclonal Antibody (VLN01), Invitrogen, 47 citations

Eukaryotic cell lines

Policy information about cell lines and Sex and Gender in Research

Cell line source(s)	Parental SNU16, COLO320-DM, H716 and HCT116 were obtained from ATCC. The TR14 neuroblastoma cell line was a gift from J. J. Molenaar (Princess Máxima Center for Pediatric Oncology, Utrecht, Netherlands). The GBM39 cell line was derived from a patient with glioblastoma undergoing surgery at Mayo Clinic, Rochester, Minnesota as described previously (PMID: 16609043). The monoclonal SNU16m1 was a subline of the parental SNU16 cells generated from a single cell after lentiviral transduction and stable expression of dCas9-KRAB as we previously described (PMID: 34819668). The CA718 cell line was derived from a patient with glioblastoma, as described previously and was obtained from the UCSD Moores Cancer Center (PMID: 28178237).
Authentication	Cell lines obtained from ATCC were not authenticated. TR-14 cell line identity for the master stock was verified by STR genotyping (IDEXX BioResearch, Westbrook, ME).
Mycoplasma contamination	Cells were tested negative for mycoplasma.
Commonly misidentified lines (See ICLAC register)	None of the cell lines used are registered by ICLAC as commonly misidentified.
