# Supplementary material for: Coordinated inheritance of extrachromosomal DNAs in cancer cells
Source: Nature. 2024 Nov 6;635(8037):201–9. doi: 10.1038/s41586-024-07861-8 (PMC11541006; doi:10.1038/s41586-024-07861-8)

1. shGFP
2. shGFP + nutlin-3a
3. shTP53
4. shTP53 + nutlin-3a

Raw image of western blot for MDM2. Image was cropped to remove extra white space. Corresponds to **Extended Data Figure 10h**.

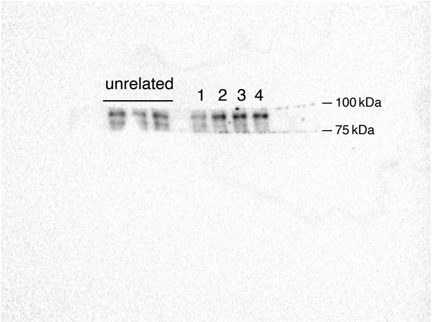

1. shGFP
2. shGFP + nutlin-3a
3. shTP53
4. shTP53 + nutlin-3a

Raw image of western blot for p53. Image was cropped to remove extra white space. Corresponds to **Extended Data Figure 10h**.

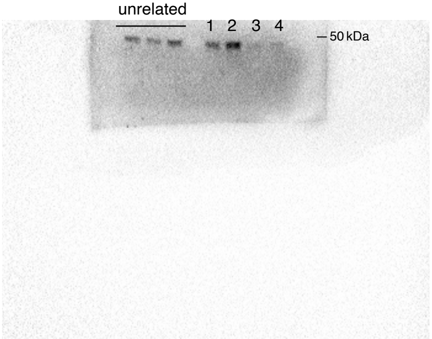

1. shGFP
2. shGFP + nutlin-3a
3. shTP53
4. shTP53 + nutlin-3a

Raw image of western blot for vinculin. Image was cropped to remove extra white space. Corresponds to **Extended Data Figure 10h**.

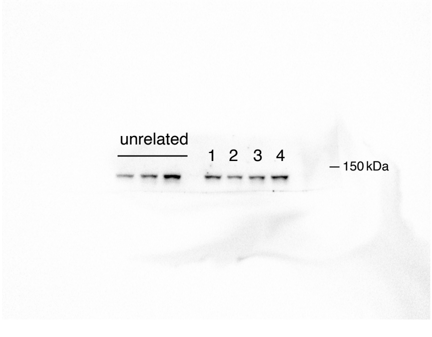

Supplement: Supplementary file 3 — Source Data Extended Data Fig. 10 [file 41586_2024_7861_MOESM3_ESM.pdf]
